# Ectomycorrhizal fungal communities in endangered *Pinus amamiana* forests

**DOI:** 10.1371/journal.pone.0189957

**Published:** 2017-12-19

**Authors:** Masao Murata, Seiichi Kanetani, Kazuhide Nara

**Affiliations:** 1 Graduate School of Frontier Sciences, The University of Tokyo, Kashiwa, Chiba, Japan; 2 Kyushu Research Center, Forestry and Forest Products Research Institute, Chuo-ku, Kumamoto, Japan; Friedrich Schiller University, GERMANY

## Abstract

Interactions between trees and ectomycorrhizal (ECM) fungi are critical for the growth and survival of both partners. However, ECM symbiosis in endangered trees has hardly been explored, complicating conservation efforts. Here, we evaluated resident ECM roots and soil spore banks of ECM fungi from endangered *Pinus amamiana* forests on Yakushima and Tanegashima Islands, Kagoshima Prefecture, Japan. Soil samples were collected from remaining four forests in the two islands. The resident ECM roots in soil samples were subjected to molecular identification. Soil spore banks of ECM fungi were analyzed via bioassays using a range of host seedlings (*P*. *amamiana*, *P*. *parviflora*, *P*. *densiflora* and *Castanopsis sieboldii*) for 6–8 months. In all remaining *P*. *amamiana* forests, we discovered a new *Rhizopogon* species (*Rhizopogon* sp.1), the sequence of which has no match amoung numerous *Rhizopogon* sequences deposited in the international sequence database. Host identification of the resident ECM roots confirmed that *Rhizopogon* sp.1 was associated only with *P*. *amamiana*. *Rhizopogon* sp.1 was far more dominant in soil spore banks than in resident ECM roots, and its presence was confirmed in nearly all soil samples examined across the major remaining populations. While *Rhizopogon* sp.1 did not completely lose compatibility to other pine species, its infection rate in the bioassays was highest in the original host, *P*. *amamiana*, the performance of which was improved by the infection. These results indicate that *Rhizopogon* sp.1 is very likely to have a close ecological relationship with endangered *P*. *amamiana*, probably due to a long co-evolutionary period on isolated islands, and to play the key role in seedling establishment after disturbance. We may need to identify and utilize such key ECM fungi to conserve endangered trees practically.

## Introduction

Forests have disappeared and deteriorated all over the world due to habitat destruction and environmental changes caused by human activity [1]. As a result, many tree species are threatened with extinction [2]. In particular, the dominant trees in a forest play critical roles in primary production and ecosystem structuring, directly and/or indirectly supporting many other organisms. Thus, the extinction of a dominant tree species can have a serious impact on biodiversity in the entire forest ecosystem, and for this reason, conservation measures are urgently needed for dominant trees threatened with extinction.

Dominant trees in temperate forests, such as species of the Fagaceae and Pinaceae, are associated with ectomycorrhizal (ECM) fungi and depend on them for soil nutrients. Without compatible ECM fungi, these trees cannot grow normally [3]. Moreover, it has become increasingly clear that the species composition and distribution of ECM fungi in soil affect the establishment of tree seedlings [4, 5]. Therefore, knowledge of the ECM fungi associated with an endangered tree species could be key to its conservation.

There are two main infection pathways used by ECM fungi in nature: the mycelium (mycelial network) extending from the existing ECM roots (e.g. [6]) and spores dispersed from fruiting bodies [7]. Both types of infection are ubiquitous in soils of developed forests, where host roots can be easily infected with ECM fungi. Mycelial networks are the predominant infection pathway in less disturbed forests, while spores that have accumulated in the soil, called soil spore banks, are the primary source of ECM infection for regenerating seedlings after disturbances that eliminate existing trees [8, 9]. Therefore, pioneer trees that require disturbance for regeneration depend on both soil spore banks, at the seedling stage, and mycelial networks, at the mature stage.

The species composition of ECM fungi often differs between spore banks and resident ECM roots, with soil spore banks usually composed of fewer ECM fungal species [8, 10–12]. Especially in pine forests, soil spore banks are dominated by pine-specific *Rhizopogon* [8, 10, 13–15], because their spores have a longer life span and greater environmental resistance than do those of other ECM fungi [16–19]. All *Rhizopogon* species produce hypogeous sporocarps and depend on animal ingestion for spore dispersal [14, 20]. Thus, gene flow of *Rhizopogon* species is more restricted than fungi producing epigeous sporocarps and is easily inhibited by geographical barriers [21]. This limited gene flow leads to genetic differentiation among isolated *Rhizopogon* populations [21–23] and eventually to speciation in geographically isolated areas [24, 25]. While most pine species are successful pioneer colonizers after disturbances throughout the northern hemisphere [20, 26], establishment of their seedlings may be sustained by unique *Rhizopogon* species that evolved locally.

The genus *Pinus* consists of two subgenera, four sections and over 100 extant species, and it is the largest genus of conifers and the most widespread genus of trees in the Northern Hemisphere [27]. While some pine species have wide distribution ranges over a continent, many have been restricted to small isolated areas [27] as a result of long biogeographic history since the Early Cretaceous [27–29] and competition with broadleaf trees. Species with small distribution ranges are prone to extinction caused by environmental and demographic stochasticity in combination with inbreeding depression [30]. In fact, 19 pine species are classified as threatened (12 endangered and 7 vulnerable species) [2].

*Pinus amamiana* is endemic to Yakushima and Tanegashima Islands, Kagoshima Prefecture, Japan [27] and is classified as “Endangered A3ce; B1ab(iii,v)+2ab(iii,v)” on the IUCN Red List and as “Vulnerable” by the Japanese government [31]. The abundance of *P*. *amamiana* has been drastically reduced by timber harvesting and pine wilt disease, and only 1,500 and 100 individuals persist in Yakushima and Tanegashima Islands, respectively [31]. Although we know nothing about the ECM fungi colonizing this endangered pine species, *P*. *amamiana* may be associated with unique ECM fungi that coevolved locally and may depend on these fungi for existence.

ECM fungi of endangered trees have long been overlooked, and thus available information on these fungi is quite limited. However, our recent study in forests of *Pseudotsuga japonica* (Pinaceae), another endangered conifer species in Japan, found that soil spore banks are dominated by *Rhizopogon togasawariana* that coevolved with the endangered host for more than 30 million years [12]. Notably, this fungus was completely absent in resident tree roots [32]. Because *P*. *japonica* is a light-demanding pioneer tree species, its seedlings are rarely found on the dark forest floor [33, 34]. These findings strongly suggest that both *P*. *japonica* and *R*. *togasawariana* depend on disturbance for regenerating their populations, and that *R*. *togasawariana* plays the key role in the establishment of seedlings during regeneration. The existence of this ECM fungus specific to the endangered tree species and its potential role in establishment of endangered tree seedlings have critical implications for conservation; yet, it is difficult to generalize the results obtained from this single case. Similar studies in *Pinus*, which includes far more endangered species than those of *Pseudotsuga*, would further improve our understanding of ECM symbiosis on endangered trees.

In the present study, our objective was to characterize the ECM fungal communities of both soil spore banks and resident ECM roots in remaining *P*. *amamiana* forests. Specifically, we examined the following hypotheses: (1) *P*. *amamiana* is associated with some host specific ECM fungi that have coevolved with this endangered pine, and (2) these fungi are more frequently detected in soil spore banks than in resident ECM roots as *Rhizopogon* species in other ecosystems. We believe that the results of this study can provide key information for *in situ* conservation of *P*. *amamiana* and has many important implications for the conservation of other endangered trees.

## Materials and methods

### Study sites

This study was conducted in four *P*. *amamiana* forests, i.e. Hirauchi (site 1) and Banri (site 2) on Yakushima Island, and Wasedagawa (site 3) and Injo (site 4) on Tanegashima Island, Kagoshima Prefecture, Japan (Table 1, S1 Appendix). Sampling permission for the site 1, 2 and 3 was issued by Yakushima forest office, and it for site 4 was issued by the owner of the land. These forests harbor the majority of remaining *P*. *amamiana* populations. At the Yakushima sites, *P*. *amamiana* is usually found on steep slopes or ridges with other ECM trees (*Castanopsis sieboldii*, *Lithocarpus edulis*, *Quercus acuta*, *Q*. *phillyraeoides*, *Q*. *salicina*, *Tsuga sieboldii*). At the Tanegashima sites, this tree is usually found on hilly land (site 3) or by the seaside (site 4) along with other ECM trees (*C*. *sieboldii*, *L*. *edulis*, *P*. *thunbergii*, *Q*. *salicina*). In addition to ECM trees, these forests also contain non-ECM sub-trees (i.e. *Cleyera japonica*, *Cryptomeria japonica*, *Distylium racemosum*, *Elaeocarpus japonicas*, *Ilex pedunculosa*, *Morella rubra*, *Myrsine seguinii*, *Pieris japonica*, *Prunus* sp., *Rhaphiolepis indica* var. *umbellate*, *Rhododendron tashiroi*, *Syzygium buxifolium*, *Ternstroemia gymnanthera*). The annual mean temperature ranged from 17.9 to 20.4°C, with abundant precipitation (Table 1).

**Table 1:** Study site characteristics.

Site	Elevation (m)	Mean annual precipitation (mm)	Annual mean temperature (°C)	Site area (ha)	Latitude/longitude	Mean DBH of *Pseudotsuga japonica* (cm)
Hirauchi (site 1)	363–514	3114	20.4	1.12	N: 30°14' 49.7–57.6", E: 130°30' 14.9–18.5"	44.2 (n = 41)
Ohko Forestry Road Banri (site 2)	635–748	2412	-	0.54	N: 30°19' 40.1–53.4", E: 130°24' 37.7–42.2"	38.4 (n = 21)
Wasedagawa (site 3)	71–96	2941	17.9	0.33	N: 30°36' 36.9–39.2", E: 131°01' 43.4–45.4"	19.7 (n = 32)
Injo (site 4)	87–103	2941	17.9	0.24	N: 30°34' 23.1–27.9", E: 131°01' 27.1–31.0"	25.8 (n = 10)

### Sampling

In September 2014, 26, 21 and 32 pairs of soil samples were collected near randomly selected *P*. *amamiana* trees at sites 1, 2 and 3, respectively. In November 2015, an additional 15 and 10 soil samples were collected at sites 1 and 4, respectively. The number of samples collected depended on the number of surviving trees at each site. Selected trees were >5 m apart. Two soil samples (5 × 5 × 10 cm depth), one for resident ECM root analysis and the other for soil spore bank analysis, were collected near each selected tree. Geographical positions were recorded using a GPS (GPSMAP62SJ, Garmin, Olathe, KS, USA). Samples were placed separately in plastic bags and kept at 4°C until further analyses.

### Bioassays

Bioassay experiments were performed to assess the soil spore banks following an established method [11, 12]. In these experiments, only soil samples collected in 2014 were used. After removing roots, organic debris and small stones, the soil samples were air-dried for 2–8 months (S2 Appendix). The bioassay containers were 50-mL centrifuge tubes (Ina Optika Inc., Osaka, Japan), with two drainage holes near the bottom blocked by a cotton ball to prevent soil loss. Each tube was filled with approximately 40 mL of the air-dried soil sample.

We used four tree species, *P*. *amamiana*, *P*. *parviflora*, *P*. *densiflora* and *Castanopsis sieboldii* (Fagaceae), to evaluate the host specificity of soil propagule banks, particularly the specificity of the *Rhizopogon* spp., which are expected in spore banks. While *P*. *parviflora* belongs to the same subgenus (*Strobus*) as that of *P*. *amamiana*, *P*. *densiflora* belongs to the subgenus *Pinus*. *C*. *sieboldii* is a broadleaf tree species that is predominant in warm-temperate forests of the region. We were able to use only 46 *P*. *amamiana* seeds for all bioassay tubes because of the limited seed availability and low germination rate of this endangered species.

Seeds of all tree species were soaked in tap water for 48 h, surface-sterilized using a 5% sodium hypochlorite solution for > 10 min, and rinsed with tap water. To induce germination, surface-sterilized *P*. *amamiana* and *P*. *parviflora* seeds were placed on sterilized, well-moistened peat moss in an incubator (25°C for 1 month and then 5°C for 2 months), while *P*. *densiflora* and *C*. *sieboldii* seeds were placed on sterilized Shibanome soil (fine red granular soil of Pleistocene volcanic origin) in an incubator (25°C). For each soil-host combination, one germinated seed was planted and covered with sterilized Shibanome soil in a bioassay tube, for a total of 283 seedlings (S2 Appendix). To monitor airborne spore contamination of ECM fungi, we prepared a control treatment using 10 tubes containing an autoclaved, randomly selected soil sample (per host). Bioassay seedlings were watered with tap water (2–3 mL per seedling) every 3–5 days depending on soil conditions and were harvested after 6–9 months of growth in a growth chamber (MLR-351; SANYO Inc. Tokyo, Japan) set to 25°C with 16 h of light (15 fluorescent lamps: 20,000 lx) and then to 20°C for 8 h of dark (S2 Appendix).

### Identification of ectomycorrhizal fungi

Roots of resident trees collected from field soil samples and bioassay seedlings were gently washed with tap water. ECM root tips were classified into morphotypes under a dissecting microscope based on their surface color, texture and emanating hyphae, as described in previous studies [35, 36]. For molecular identification of ECM fungi, triplicate ECM root tips, if available, were selected for each morphotype in a soil sample, then placed individually into 2.0-mL tubes. A total of 682 root tips from ECM were used for molecular identification on resident trees. In addition, 208, 246, 257 and 191 ECM root tips from the bioassays of the *P*. *amamiana*, *P*. *parviflora*, *P*. *densiflora* and *C*. *sieboldii*, respectively, were subjected to DNA analysis.

Molecular identification of ECM fungi was performed following Murata et al. [12]. Briefly, each ECM tip sample was placed in a 2.0-mL tube containing a zirconia ball and then pulverized using a bead beater. Total DNA was extracted using a modified cetyl trimethylammonium bromide method [35]. Internal transcribed spacer regions (ITS1-5.8S-ITS2) of ribosomal DNA were amplified using the ITS1F or ITS0F-T forward primer and several reverse primers (ITS4, ITS4Cg, LB-W [37–40] depending on amplification success. Platinum® Multiplex PCR Master Mix (Applied Biosystems, Foster City, CA, USA) was used for polymerase chain reaction (PCR).

The amplified products were purified and subjected to direct sequencing (3730xl DNA Analyzer; Applied Biosystems) using ITS1 as the sequencing primer. For poorly sequenced samples, ITS4 was also used as a sequencing primer. The obtained high-quality sequences were grouped into molecular operational taxonomic units (MOTUs) with ≥ 97% ITS sequence similarity (including 5.8S regions) using ATGC software (ver. 7.0; GENETYX Corp., Tokyo, Japan). Species identity was assigned based on the results of BLAST searches against known taxa in international sequence databases (DNA Data Bank of Japan [DDBJ]/EMBL/GenBank). Potential PCR chimeras were removed manually before further analyses, based on inconsistent BLAST results between segments within each MOTU sequence, e.g., between the initial and last parts (about 100 bp each) of the sequence. If the ITS similarity with a described species from herbarium specimens was > 97%, we used that species name for the MOTU. When the similarity to known species was 90–97% or < 90%, we identified MOTUs at the genus and family level, respectively. One exception was *C*. *geophilum*, which was identified by amplifying the *Cenococcum*-specific primer (ITS4Cg) and confirmed by sequencing randomly selected samples. Although several different MOTUs of *C*. *geophilum* were found (< 97% ITS sequence similarity), they were treated as one MOTU in this study, because no taxonomic agreement was reached among the many previous studies of ECM fungal communities (e.g., [36, 41]). Representative sequences of individual MOTUs were deposited in the DDBJ under accession numbers LC315810–LC315919.

To confirm the identity of host species in all molecular samples, the plastid trnL or rbcL region of plant DNA was amplified using primers trnC (5′-cgaaatcggtagacgctacg-3′) or rbcLa-F (5′- atgtcaccacaaacagagactaaagc- 3′) in combination with trnD (5′-ggggatagagggacttgaac- 3′) or rbcLa-R (5′- gtaaaatcaagtccaccrcg- 3′). All the amplicons were purified and subjected to direct sequencing using trnC or rbcLa-F as the sequencing primer. As with species identification of ECM fungi, the identity of host species was assigned based on the results of BLAST searches against known taxa in international sequence databases.

### Statistical analyses

The frequency of an ECM fungal species was defined as the number of bioassay seedlings colonized by each ECM fungal species or, for resident trees, the number of soil samples containing each ECM fungal species. The frequencies of major fungal species were compared between different bioassay host species using Fisher’s exact test implemented in SPSS (ver. 11.5; SPSS Japan Inc., Tokyo, Japan). Statistical significance was set at *α* = 0.05. Estimate S software (ver. 8.2; [42]) was used to calculate the minimum number of total species richness indicators (Jackknife 2).

A fungal community matrix was built for the major host groups (*P*. *amaiana*, *T*. *sieboldii* and Fagaceae) at each site. Community compositional similarity was visualized using non-metric dimensional scaling (NMDS), implemented in the ‘vegan’ package of R software [43], using the Bray-Curtis distance (Manhattan distance for NMDS based on the unit of soil samples for resident ECM roots) and 999 permutations. Differences in community composition between resident ECM roots and assayed spore banks were determined using Adonis (permutation multivariate analysis of variance; [44]) in ‘vegan’, again using the Bray-Curtis distance and 999 permutations [45]. Data dispersions of the communities among groups were analyzed by the Betadisper test (Permutational analysis of multivariate dispersions; [44]) in ‘vegan’.

## Results

### ECM fungal communities of *Pinus amamiana* forests

Of the 104 soil samples collected from the four study sites, 44 contained *P*. *amamiana* ECM roots. Other dominant host taxa in the belowground ECM tips were *T*. *sieboldii* and Fagaceae, which were found in 9 and 58 soil samples, respectively (Table 2). As a minor host, the subgenus *Pinus* was confirmed in one soil sample. No ECM roots were found in 15 soil samples.

**Table 2:** Ectomycorrhizal (ECM) fungal species detected on resident tree roots from *Pinus amamiana* forests. The number of soil samples containing each ECM fungus is shown, along with sequence accession numbers and BLAST results.

Experiment types	Among-forests comparison				Host ^a^	DDBJ accession No.	Seq. length (bp)	Best BLAST match		
Sites	1	2	3	4						
ECM fungi								Accession No.	Query cov.	Max. ident.
*Amanita* sp.1	0	1	0	0	T		559	KP711844	98	96
*Amanita* sp.2	1	0	0	0	P		639	AB922858	99	99
*Amanita* sp.3	0	1	0	0	P		688	KC424544	100	99
*Amanita* sp.4	0	0	1	0	F		203	JQ991635	100	96
Amanitaceae sp.1	1	0	0	0	F		393	KP276311	89	89
*Austroboletus* sp.1	1	0	0	0	P, T		616	JQ991650	91	99
*Boletellus aurocontextus*	0	0	1	1	F		798	AB989004	100	99
*Boletus* sp.1	1	1	0	0	F		639	AB973752	100	99
*Boletus* sp.2	0	0	1	0	F		694	KX756398	66	90
*Boletus* sp.3	0	0	1	0	-		644	AB973811	81	99
Boletaceae sp.1	1	5	0	0	P, T		439	AB972824	99	100
Boletaceae sp.2	1	0	4	0	F		794	KM198314	93	83
Boletaceae sp.3	0	1	0	0	F		692	KJ676960	100	92
Boletaceae sp.4	0	1	0	0	F		262	KC552019	100	94
Boletaceae sp.5	0	1	1	0	F		806	KC551993	100	99
Boletaceae sp.6	0	2	0	0	P		765	AB973727	100	99
Boletaceae sp.7	0	0	1	0	P		630	AB509871	59	99
Boletaceae sp.8	1	0	0	0	P		652	KM595001	99	92
Boletaceae sp.9	0	1	0	0	F		572	JQ991917	100	100
Boletaceae sp.10	0	0	0	1	F		675	JF273511	100	99
Cantharellaceae sp1	1	0	0	0	F, T		565	KT200524	84	93
*Cenococcum geophilum*	12	6	15	0	F, P, T	-	-	-	-	-
Ceratobasidiaceae sp.1	0	1	0	1	P, T		584	AB605643	100	100
Ceratobasidiaceae sp.2	0	1	0	0	P		597	AB605649	98	96
*Ceratobasidium* sp.1	0	0	0	1	P		612	AB303058	100	99
*Ceratobasidium* sp.2	0	0	0	1	P		602	JQ991676	93	98
*Clavulina* sp.1	0	0	0	1	F		569	JF273519	100	100
Clavulinaceae sp.1	2	0	0	0	F, P, T		641	AM412265	100	89
Clavulinaceae sp.2	2	0	0	0	P, T		711	AB807913	92	99
Clavulinaceae sp.3	5	0	0	0	F, P, T		403	AB807910	94	99
Clavulinaceae sp.4	1	0	0	0	P		655	KC876295	100	87
Clavulinaceae sp.5	0	1	0	0	P		592	AB848424	100	100
Clavulinaceae sp.6	0	1	0	0	F		235	KC876295	100	89
Clavulinaceae sp.7	1	0	0	0	P		601	FR731325	100	88
*Coltricia* sp.1	1	0	0	0	P		268	KU360702	100	95
*Cortinarius* sp.1	0	0	2	0	F, P		737	HQ604690	100	93
*Cortinarius* sp.2	0	0	1	0	F		616	AB848438	100	99
*Cortinarius* sp.3	0	0	1	0	F		660	LC096896	100	92
*Cortinarius* sp.4	0	1	0	0	F		780	AB973753	100	99
*Cortinarius* sp.5	0	0	0	1	F, P		507	HQ285384	100	96
*Craterellus* sp.1	0	1	0	0	F		237	JQ991672	99	94
*Elaphomyces* sp.1	1	0	3	0	F		535	JQ991717	92	95
*Elaphomyces* sp.2	0	1	0	0	F		417	KX165351	98	97
Elaphomycetaceae sp.1	0	0	1	0	F		337	JQ991901	99	98
*Entoloma* sp.1	0	0	1	0	F		554	KP403072	100	97
*Hydnellum* sp.1	0	0	1	0	F		602	EU293832	84	92
*Hydnellum* sp.2	0	0	1	0	P		601	KM403015	71	97
*Hydnum* sp.1	1	0	0	0	P		555	KU612573	94	99
*Hydnum* sp.2	0	0	1	0	F		569	AB906676	100	100
*Hydnum* sp.3	1	0	0	0	P		392	AB251813	100	100
Hymenochaetaceae sp.1	0	1	0	0	P		704	JQ991687	91	89
Hymenochaetaceae sp.2	1	0	0	0	P		710	KM594897	99	91
*Inocybe* sp.1	1	0	0	0	T		528	AM882711	100	95
*Lactarius* sp.1	2	1	0	0	F		625	AB777482	100	99
*Lactarius* sp.2	0	0	2	0	F, P		654	LC096473	100	99
*Lactarius* sp.3	1	0	0	0	F		638	JQ991640	96	99
*Lactarius* sp.4	0	1	0	0	T		656	GQ268638	100	98
*Lactarius* sp.5	0	1	0	0	F		573	AB973742	100	99
*Lactarius* sp.6	0	1	0	0	F		543	JQ991763	97	94
Pezizaceae sp.1	0	1	0	0	F		565	JN102406	99	94
Pezizaceae sp.2	0	0	0	1	F		360	JQ991767	100	95
*Phylloporus* sp.1	7	1	0	0	F, P, T		835	DQ533980	99	91
*Phylloporus* sp.2	0	1	2	0	F		775	AB973776	82	99
*Phylloporus* sp.3	0	1	1	0	F		765	JQ967270	82	96
*Rhizopogon* sp.1	5	3	1	2	P		754	LC216339		
*Rhizopogon* sp.2	0	0	0	1	Ps		286	AB923020	99	99
*Rossbeevera griseovelutina*	0	1	0	0	F		505	KC551986	100	99
*Russula* sp.1	3	0	6	0	F, P		768	AB507012	84	99
*Russula* sp.2	0	0	2	0	F		600	JX556180	99	99
*Russula* sp.3	0	0	1	0	F, P		625	JX987768	99	99
*Russula* sp.4	2	0	0	0	F, P		608	LC096863	100	99
*Russula* sp.5	1	0	0	0	F		641	JQ991802	94	99
*Russula* sp.6	0	0	1	0	F		687	AB507006	88	99
*Russula* sp.7	1	0	0	0	F		712	LC096934	100	96
*Russula* sp.9	1	0	0	0	F		488	AB509875	82	99
*Russula* sp.10	0	1	0	0	P		630	AB594957	94	99
*Russula* sp.11	0	0	1	0	-		526	EF634135	100	90
*Russula* sp.12	0	0	1	0	F		573	AB629011	100	99
*Russula* sp.13	0	0	1	0	-		535	AB636110	97	99
*Russula* sp.14	0	0	1	1	F, P		718	LC096811	100	100
*Russula* sp.15	1	1	0	1	F, P		736	KJ769295	99	99
*Russula* sp.16	1	0	0	0	-		502	JN129409	100	96
*Russula* sp.17	0	0	0	1	F		608	AB291756	98	99
*Russula* sp.18	0	0	0	1	F		603	JQ991823	97	99
*Russula* sp.19	1	0	0	0	F		615	LT602972	99	97
*Russula* sp.20	1	0	0	0	F		614	JQ991790	99	99
*Russula* sp.21	1	0	0	0	P		594	AB769909	100	95
*Russula* sp.23	1	0	0	0	F		599	AB218154	100	99
*Russula* sp.24	0	0	0	1	P		297	AB973714	100	97
Russulaceae sp.1	1	0	0	0	-		623	GQ268654	99	94
Russulaceae sp.2	1	0	0	0	F		513	KM594995	100	95
*Sarcodon* sp.1	0	0	2	0	F		592	KR673603	100	95
*Sistotrema* sp.1	0	0	1	0	F		530	JX561240	100	94
Thelephoraceae sp.1	3	2	0	0	P		703	JF519143	100	97
Thelephoraceae sp.2	0	0	1	0	F		608	JF273549	100	99
Thelephoraceae sp.3	0	0	0	1	P		624	KM403070	100	96
Thelephoraceae sp.4	1	1	0	0	P		589	FJ210778	100	95
Thelephoraceae sp.5	1	1	0	0	P		594	AB854719	98	98
Thelephoraceae sp.6	0	1	0	0	T		751	EU292520	100	96
*Tricholoma* sp.1	0	0	0	1	F		620	LT000094	100	98
*Tuber* sp.1	1	0	0	0	P		522	AB285533	100	98

^a^ F, P, Ps and T indicate Fagaceae, *Pinus amamiana*, *Pinus* sp. (subgenus *Pinus*) and *Tsuga sieboldii*, respectively.

**Fig 1:**
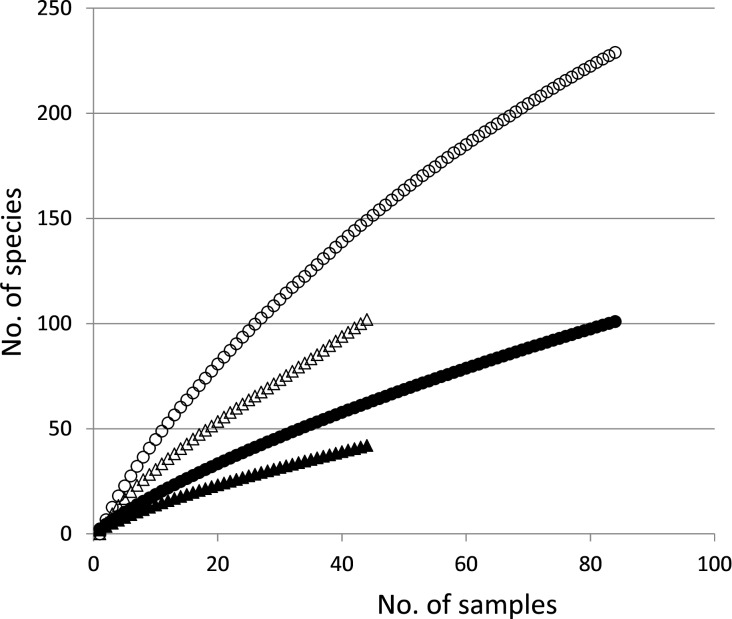
Sample-based rarefaction curves for ectomycorrhizal (ECM) fungi found in *Pinus amamiana* forests. Black circles and triangles represent observed ECM fungal species richness for all host species and for *P*. *amamiana*, respectively. Jackknife2 minimal species richness estimates of ECM fungi are also shown for all host species (white circles) and for *P*. *amamiana* (white triangles).

Of the 101 putative ECM fungi that were identified in the four sites, 42 species were found on *P*. *amamiana* roots (Table 2, S3 Appendix). Thirteen and sixty-one ECM fungi were found on *T*. *sieboldii* and Fagaceae roots, respectively. The rarefaction curves indicated that neither the observed ECM fungal richness on *P*. *amamiana* nor richness on all hosts approached an asymptote with the maximum sample size (Fig 1), indicating that additional fungal species would be found with additional sampling effort. The richness estimator Jackkife2 showed that at least 229 ECM fungal species should inhabit these forests (Fig 1). The Jackkife2 richness estimator for ECM fungi on *P*. *amamiana* was 102 (Fig 1). The observed ECM fungal richness Jackkife2 estimators on *T*. *sieboldii* and Fagaceae trees were 31 and 141, respectively.

The total ECM fungal community was composed of a few common species and a large number of rare species. Only eight ECM fungal taxa appeared in five or more soil samples. In contrast, 73 taxa were found only once (i.e., singletons). *Cenococcum geophilum* was the most frequent taxon, found in 39% of the soil samples. The occurrence frequencies of Russulaceae (48%), Boletaceae (36%), Clavulinaceae (17%), Rhizopogonaceae (14%) and Thelephoraceae (13%) were also high at the family level. Russulaceae (30 spp.) was the most species-rich ECM fungal lineage, followed by Boletaceae (19 spp.), Clavulinaceae (eight spp.) and Thelephoraceae (six spp.), while only two species of Rhizopogonaceae were found (Table 2).

Only *Rhizopogon* sp.1 was found at all sites (Table 2). In addition, Boletaceae sp.1, Clavulinaceae sp.3, *Phylloporus* sp.1 and Thelephoraceae sp.1, among the major ECM fungal species with >5% relative frequencies, were only found on Yakushima Island (sites 1 and 2). In contrast, none of the major ECM fungi were exclusive to Tanegashima Island (sites 3 and 4).

**Fig 2:**
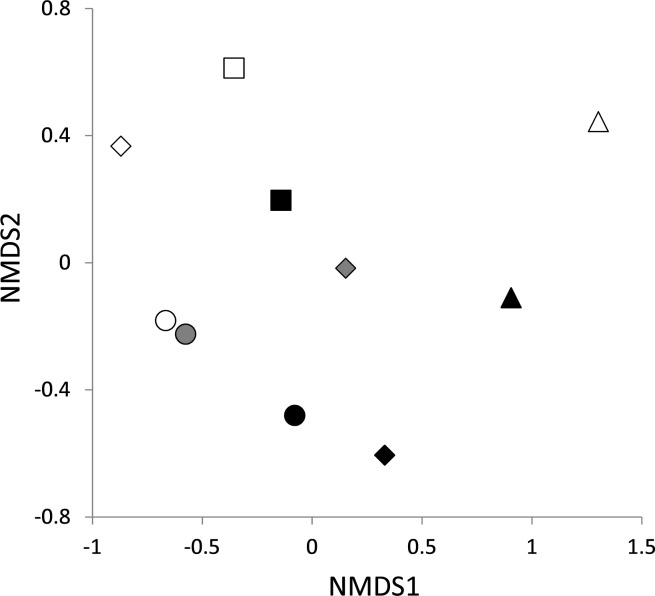
Non-metric multidimensional scaling (NMDS) depicting ECM fungal communities of resident trees in four endangered *Pinus amamiana* forests. Stress = 0.089. White, gray and black symbols indicate the communities on *P*. *amamiana*, *Tsuga sieboldii* and Fagaceae, respectively. Circles, diamonds, squares and triangles represent communities at sites 1, 2, 3 and 4, respectively.

*Cenococcum geophilum*, Clavulinaceae sp.1, Clavulinaceae sp.3 and *Phylloporus* sp.1 were found in all of the host groups (two conifer species and Fagaceae, Table 2). In contrast, *Rhizopogon* sp.1 and Thelephoraceae sp.1 were only found in *P*. *amamiana*, with a >5% relative frequency, while Boletaceae sp.2, *Elaphomyces* sp.1, *Lactarius* sp.1 and *Phylloporus* sp.2 were only found in Fagaceae. ECM fungal communities were separated by host groups (Fig 2, S4 Appendix), with statistical significance, as determined by the Adonis test (*pseudo-F*_*2*,*4*_ = 2.06, *R*^*2*^ = 0.26, *P* < 0.01) in combination with the Betadisper test (*F*_,*2*, *7*_ = 3.241, *P* = 0.101). The effect of site was also significant by the Adonis test (*pseudo-F*_*3*,*4*_ = 2.56, *R*^*2*^ = 0.49, *P* < 0.01), yet it was affected by the difference in data dispersion (Betadisper test: *F*_*3*,*6*_ = 8.484, *P* = 0.014).

### Bioassay experiments

By the end of the growth period, three, fifteen, two and seven seedling mortalities occurred among the *P*. *amamiana*, *P*. *parviflora*, *P*. *densiflora* and *C*. *sieboldii* soil samples, respectively (S5 Appendix). ECM formation was observed in 100%, 81.3%, 75.3% and 80.6% of seedlings in *P*. *amamiana*, *P*. *parviflora*, *P*. *densiflora* and *C*. *sieboldii*, respectively. Five, four, nine and seven ECM fungi were identified from 208 *P*. *amamiana*, 246 *P*. *parviflora*, 257 *P*. *densiflora* and 191 *C*. *sieboldii* DNA samples, respectively (Table 3 and S3 and S5 Appendices). None of the control seedlings had ECM roots.

**Table 3:** Ectomycorrhizal (ECM) fungal species detected from soil spore banks in *Pinus amamiana* forests by bioassay experiments using four host species. The number of seedlings colonized by each ECM fungus is shown, along with sequence accession numbers and BLAST results.

Experiment types	Among-forests comparison												DDBJ accession No.	Seq. length (bp)	Best BLAST match		
Bioassay hosts ^a^	Pa			Pp			Pd			C							
Sites	1	2	3	1	2	3	1	2	3	1	2	3			Accession No.	Query cov.	Max. ident.
Atheliaceae sp.1	0	0	0	0	0	0	0	0	1	0	0	0		652	AB456674	84	99
*Cenococcum geophilum*	6	2	10	8	8	18	14	8	29	14	16	26	-	-	-	-	-
*Clavulina* sp.2	0	0	0	0	0	0	0	0	1	0	0	0		473	JQ991682	100	99
Clavulinaceae sp.3	0	0	0	1	0	0	1	0	0	1	0	0		403	AB807910	94	99
*Craterellus* sp.2	1	0	0	1	0	0	1	0	0	0	0	0		516	AB973729	100	98
*Elaphomyces* sp.2	0	0	0	0	0	0	0	1	0	0	0	0		417	KX165351	98	97
*Laccaria vinaceoavellanea*	0	0	0	0	0	0	0	0	0	1	0	0		480	KJ609167	100	99
Pezizaceae sp.3	0	0	0	0	0	0	0	0	0	0	0	3		539	AB571493	98	100
*Rhizopogon* sp.1	17	6	17	4	16	22	3	5	16	0	0	0		754	LC216339		
*Rhizopogon* sp.2	0	0	0	0	0	0	1	0	2	0	0	0		286	AB923020	99	99
*Rhizopogon* sp.3	1	0	0	0	0	0	0	0	0	0	0	0		541	AB253521	100	98
*Russula* sp.25	0	0	0	0	0	0	0	0	0	0	0	1		537	HE814113	100	99
*Suillus bovinus*	0	0	0	0	0	0	1	0	0	0	0	0		720	AB571499	100	99
Thelephoraceae sp.7	2	0	0	0	0	0	0	0	0	1	0	1		608	JX456851	100	99
Thelephoraceae sp.8	0	0	0	0	0	0	0	0	0	0	0	1		597	GU907787	100	96

^a^ Pa, Pp, Pd and C indicate *Pinus amamiana*, *P*. *parviflora*, *P*. *densiflora* and *Castanopsis sieboldii*, respectively.

**Fig 3:**
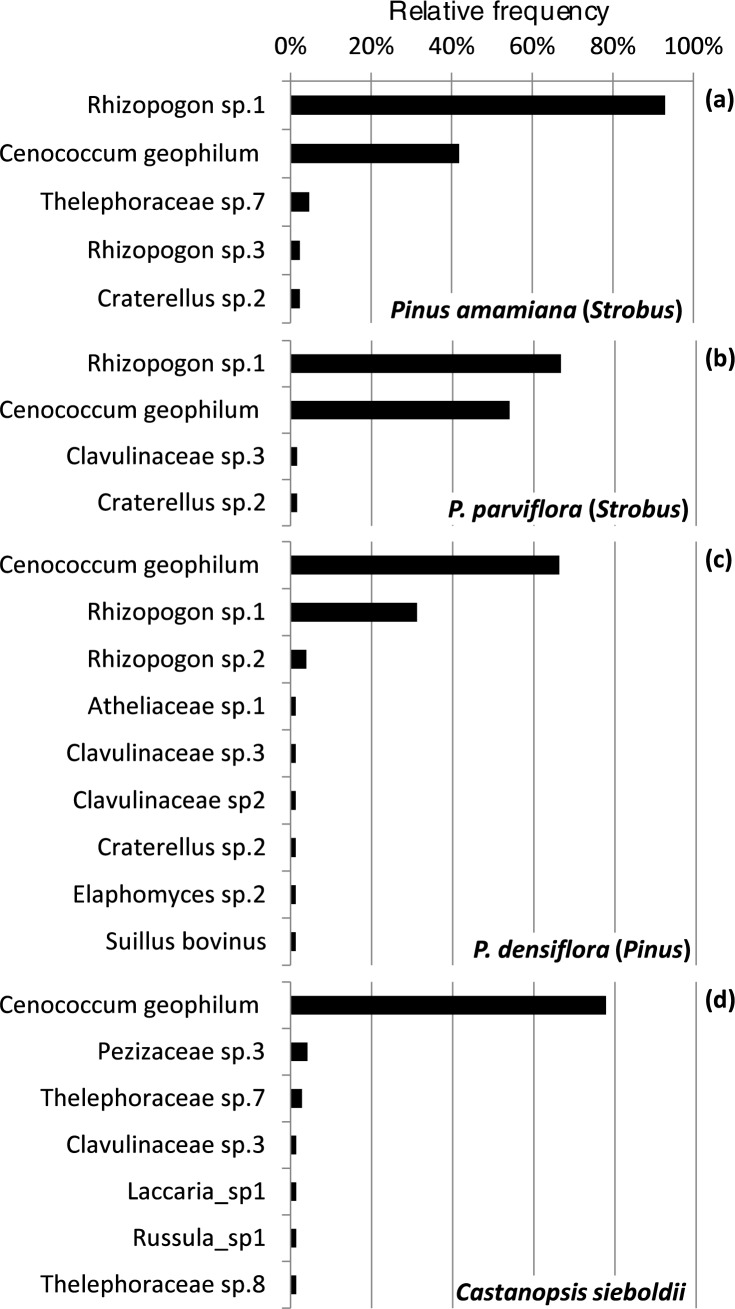
Soil propagule banks of ECM fungi in endangered *Pinus amamiana* forests assayed in four hosts: *P*. *amamiana* (a), *P*. *parviflora* (b), *P*. *densiflora* (c) and *Castanopsis sieboldii* (d). The relative frequency indicates the proportion of soil samples containing each ECM species out of all soil samples examined (S5 Appendix).

In bioassays of the subgenus *Strobus*, six ECM fungal species were found, of which three were shared between *P*. *amamiana* and *P*. *parviflora* (Table 3, Fig 3). Nine and seven ECM fungal species were detected in the *P*. *densiflora* and *C*. *sieboldii* bioassays, respectively. The estimated species richness (Jackknife2) was 7.9, 7.9, 20.7 and 13.8 in *P*. *amamiana*, *P*. *parviflora*, *P*. *densiflora* and *C*. *sieboldii*, respectively.

*Rhizopogon* sp.1 was the most dominant ECM fungal species detected in the *P*. *amamiana* bioassay, found in 40 of 43 (93% frequency) soil samples (Fig 3). This fungus was also found in *P*. *parviflora* and *P*. *densiflora* samples but at lower frequencies, 67% and 31%, respectively (Fig 3). *Rhizopogon* sp.1 was not found in any of the *C*. *sieboldii* bioassays. *Cenococcum geophilum* was the most common fungal species in the *P*. *densiflora* and *C*. *sieboldii* bioassays (Fig 3). Among the 16 ECM fungal species found in bioassays, only *C*. *geophilum* was shared among the four host species (Fig 3).

**Fig 4:**
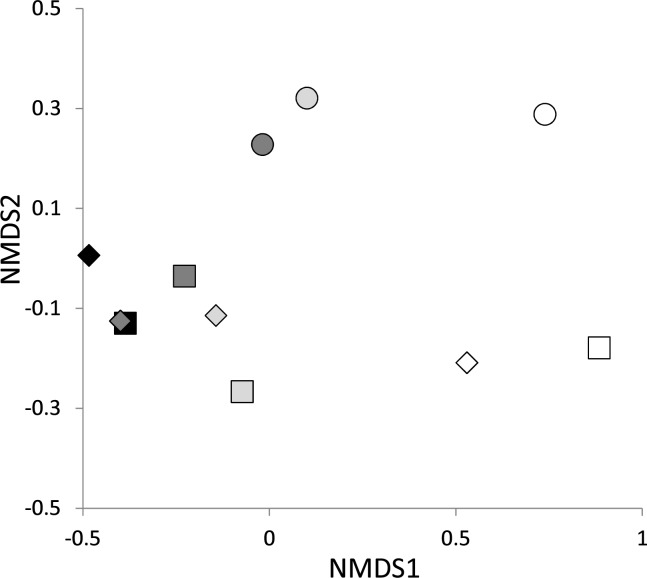
Non-metric multidimensional scaling (NMDS) depicting soil propagule bank communities of ECM fungi in three endangered *Pinus amamiana* forests. Stress = 0.066. Black, light gray, dark gray and white symbols indicate the communities assayed with *P*. *amamiana*, *P*. *parviflora*, *P*. *densiflora* and *Castanopsis sieboldii*, respectively. Circles, diamonds, and squares represent communities assayed with soils from sites 1, 2, and 3, respectively.

Soil spore bank communities were clearly separated by host and site using NMDS ordination (Fig 4), with statistical significance determined by the Adonis test (host: *pseudo-F*_*3*,*6*_ = 12.08, *R*^*2*^ = 0.69, *P* < 0.01; site: *pseudo-F*_*2*,*6*_ = 5.12, *R*^*2*^ = 0.2, *P* < 0.01) in combination with the Betadisper test (host: *F*_*3*,*8*_ = 1.351, *P* = 0.325; site: *F*_*2*,*9*_ = 1.819, *P* = 0.206). A significant difference (S6 Appendix; *pseudo-F*_1,18_ = 5.76, *R*^2^ = 0.24, *P* < 0.01) was found between soil propagule banks and resident ECM fungal communities on mature trees in these same three forests, however, it was affected by significant difference in data dispersion among the groups (Betadisper, *F*_*1*,*18*_ = 44.257, *P* < 0.001).

## Discussion

We found *Rhizopogon* sp.1 in all remaining *P*. *amamiana* forests. Host identification of the resident ECM roots confirmed that *Rhizopogon* sp.1 was associated only with the endangered species *P*. *amamiana*. In soil spore banks, *Rhizopgon* sp.1 was far more dominant than in resident ECM roots and was identified in nearly all soil samples examined (93%) among the major remaining *P*. *amamiana* populations. These findings generally support both our hypotheses, confirming the existence of an ECM fungus specific to the endangered pine species in its natural settings and the predominance of this fungus in soil spore banks [12]. *Rhizopogon* sp.1 is very likely to have a close ecological relationship with the endangered *P*. *amamiana*, probably due to long co-evolutionary periods on isolated islands.

Dominant ECM fungi in soil spore banks generally contribute to establishment of tree seedlings, especially for pine trees regenerating after a disturbance [46–48]. Thus, the *Rhizopogon* sp.1 found in this study likely also contributes to seedling establishment of the endangered *P*. *amamiana* after a disturbance [49, 50]. Although no other native pine species are distributed in the remaining forests of *P*. *amamiana*, some broadleaf trees could compete with *P*. *amamiana* during regeneration. It should be noted here that the dominant broadleaf tree *C*. *sieboldii* was not compatible with *Rhizopogon* sp.1 in the bioassay experiments. Alternatively, *C*. *sieboldii* bioassay seedlings were dominated by the true generalist *C*. *geophilum*, sclerotia of which exist in almost all forests investigated (e.g. [8, 11, 12, 15]). Murata & Nara [51] suggested that *C*. *geophilum* competes with other ECM fungi, thus affecting the infection rates of other soil spore bank fungi. While ECM symbiosis is a prerequisite for trees to grow and survive in nature [3], controlling ECM fungal communities may be difficult, especially after forest development. Eliminating other ECM fungi from soil spore banks in disturbed sites via treatments, such as heating [12, 52–54] or filtering [51], may allow an increase in the relative frequency of *Rhizopogon* sp.1 and eventually increase regeneration of the endangered *P*. *amamiana* preferentially.

We found no sequences that match *Rhizopogon* sp.1 in an international nucleotide sequence database (DNA Data Bank of Japan [DDBJ]/EMBL/GenBank), potentially indicating its endemism to these islands. In fact, based on the morphological characteristics of the sporocarps and phylogenetic relationships with other described *Rhizopogon* species, we are now describing it as a new species, which does not belong to any subgenus proposed by Grubisha et al. [55] (Sugiyama et al. under revision). The existence of a *Rhizopogon* fungus specific to an endangered tree was documented in our recent study of *Pseudotsuga* species in Japan and China; i.e., *P*. *japonica* is associated with *R*. *togasawariana* [12, 32] and *P*. *sinensis* with an undescribed *Rhizopogon* species [56]. Here, we first confirmed the existence of a *Rhizopogon* fungus specifically associated with endangered *Pinus*, which is a far larger genus than *Pseudotsuga* and includes far more endangered species. Given the predominance of the host-specific fungus in soil spore banks and its potential roles in seedling regeneration, such fungi should be explored further in other endangered *Pinus* species that have been geographically isolated for long periods. Without identifying such key ECM fungi, conservation of endangered pine species will be difficult.

*Rhizopogon* sp.1 was associated solely with the endangered *P*. *amamiana* under natural settings. This fungus was also compatible with *P*. *parviflora* and *P*. *densiflora* in bioassays, but the colonization frequency was reduced significantly with increasing phylogenetic distance. *P*. *amamiana* is closely related to *P*. *armandii* var. *armandii* and *P*. *armandii* var. *mastersiana* [57, 58], belonging to the same clade of subsection *Strobus* found in East Asian subtropical areas [29, 57], as the divergence of *P*. *amamiana* from *P*. *parviflora* or *P*. *densiflora* dates approximately 6.4 Mya or 85 Mya, respectively [29]. The partial compatibility with these distantly related pine species may indicate that the isolation period has not been long enough for compatibility to be lost completely. In previous studies of *R*. *togasawariana* associated with *Pseudotsuga japonica*, colonization in *P*. *densiflora* was totally absent in a similar bioassay, but it was compatible with North American *Pseudotsuga menziesii* [12]. *R*. *togasawariana* belongs to the subgenus *Villosuli*, which is specific to *Pseudotsuga*, and its origin precedes the migration of the host to Asia approximately 34 Mya. Although *Rhizopogon* is regarded as a host-specific ECM fungal lineage [59], its compatibility is not strict within the same host genus. Yet, the compatibility with non-native hosts may not be fully functional in terms of nutrient transfer and host performance.

Another interesting finding of this study is that *P*. *amamiana* was not associated with any *Suillus* species, either in ECM roots of resident trees or in soil spore banks. *Suillus* produces epigeous sporocarps for spore dispersal by wind but is a phylogenetic sister to *Rhizopogon* [55]. While the life span of *Suillus* spores is shorter than that of *Rhizopogon*, it is frequently recorded in soil spore banks [20], probably because of its strong spore dispersal abilities [60, 61] and good germination rates [62]. Some *Suillus* species are specific to, or prefer, the subgenus *Strobus* within the genus *Pinus* [63–65], and these fungi have wide intercontinental distributions. For example, *Suillus spraguei* is found in *Strobus* pine forests from North America to East Asia [63]. In Japan, *S*. *spraguei* is associated with *P*. *parviflora*, *P*. *pumila and P*. *koraiensis* [64, 66], all of which belong to the subgenus *Strobus*. In addition, *S*. *spraguei* and some closely related species are associated with *P*. *armandii*, which is closely related to *P*. *amamiana* [63, 67]. The nearest pine population belonging to *Strobus*, *P*. *parviflora*, is located in the Takakumayama mountains on the main island of Kyushu, which is its southern population limit [68], and this population is located >100 km away from the remaining *P*. *amamiana* forests by sea. Therefore, the absence of *Suillus* species in the remaining *P*. *amamiana* forests may indicate the difficulty of airborne spore dispersal from distant *Strobus* forests together with local extinction on Yakushima and Tanegashima Islands.

In the bioassay experiment, it was difficult to characterize the effect of ECM infection on the initial growth of seedlings, because the experimental period was too short and the bioassay tubes were too small to evaluate the growth of *P*. *amamiana*. Therefore, we did not compare the growth of seedlings infected with ECM fungi with that of control plants in the bioassays. Instead, seedlings infected with *Rhizopogon* sp.1 and uninfected seedlings were raised for 1 year in a separate bioassay experiment, after which growth was compared. Seedlings infected with *Rhizopogon* sp.1 exhibited increased growth compared with control seedlings, with marginal statistical significance (S7 Appendix) and the difference would become much larger with the growth period. Moreover, *P*. *amamiana* seedlings colonized by *Rhizopogon* sp.1 are much more tolerant to transplantation than are uncolonized seedlings (S8 Appendix). Thus, even in a nursery setting, the application of *Rhizopogon* sp.1 is a promising approach to producing good seedlings for transplantation to conserved areas.

## Supporting information

S1 Appendix Location of four study sites in Japan. (PDF)

S2 Appendix Air-dried periods, growth periods and number of sample of each tree species in bioassay experiment. (PDF)

S3 Appendix Blast results of ectomycorrhizal fungal species identified in this study using UNITE database. (PDF)

S4 Appendix Non-metric multidimensional scaling (NMDS) depicting ECM fungal communities of resident trees in four endangered *Pinus amamiana* forests based on individual soil samples. Stress = 0.142. White, gray and black symbols indicate the communities on Fagaceae, *P*. *amamiana* and *Tsuga sieboldii*, respectively. Circles, squares, diamonds and triangles represent communities at sites 1, 2, 3 and 4, respectively. The effects of both host and site were significant (Adonis, *P*<0.01) after confirming data variance among the groups was not significant (Betadisper, *P*>0.05).
(PDF)

S5 Appendix Summary of bioassays using soil from three endangered *Pinus amamiana* forests. (PDF)

S6 Appendix Comparison of ectomycorrhizal fungal communities between soil propagule banks and resident trees in endangered *Pinus amamiana* forests using non-metric dimensional scaling (NMDS). Each symbol represents a community in each host per site. Stress = 0.134. White and black symbols indicate the communities assayed with resident trees and soil propagule banks, respectively.
(PDF)

S7 Appendix In the bioassay experiment different from this study (unpublished data), the number of leaves and tree height of seedlings infected with *Rhizopogon* sp.1 and seedlings not infected with ECM fungi (control) grown for 1 year. Significant probability between *Rhizopogon* sp.1 and Control of leaf number and tree height by T test was p = 0.08 and p = 0.06, respectively. The bar shows the standard error.
(PDF)

S8 Appendix From a bioassay experiment separate from this study (unpublished data), transplantation of seedlings infected with *Rhizopogon* sp.1 (upper) and uninfected seedlings (lower) to new pots after 1 year of growth. (PDF)

## References

[1] FAO. Global Forest Resouces Assessment 2010. FAO, Italy, Rome. FAO forestry paper 163. 2010.

[2] IUCN. IUCN Red List of Threatened Species 2016 [cited 2016 July]. Available from: www.iucnredlist.org.

[3] Smith SE, Read DJ. Mycorrhizal symbiosis. Third Edition ed. London: Academic Press; 2008.

[4] Nara K. Ectomycorrhizal networks and seedling establishment during early primary succession. New Phytol. 2006;169:169–78. doi: 10.1111/j.1469-8137.2005.01545.x. ISI:000233530400017. 16390428 10.1111/j.1469-8137.2005.01545.x

[5] Nara K. Pioneer dwarf willow may facilitate tree succession by providing late colonizers with compatible ectomycorrhizal fungi in a primary successional volcanic desert New Phytol. 2006;171:187–98. doi: 10.1111/j.1469-8137.2006.01744.x 16771994 10.1111/j.1469-8137.2006.01744.x

[6] Simard SW, Beiler KJ, Bingham MA, Deslippe JR, Philip LJ, Teste FP. Mycorrhizal networks: mechanisms, ecology and modelling. Fungal Biol Rev. 2012;26:39–60.

[7] Ishida TA, Nara K, Tanaka M, Kinoshita A, Hogetsu T. Germination and infectivity of ectomycorrhizal fungal spores in relation to their ecological traits during primary succession. New Phytol. 2008;180:491–500. doi: 10.1111/j.1469-8137.2008.02572.x. ISI:000259526300022. 18657211 10.1111/j.1469-8137.2008.02572.x

[8] Taylor DL, Bruns TD. Community structure of ectomycorrhizal fungi in a *Pinus muricata* forest: minimal overlap between the mature forest and resistant propagule communities. Mol Ecol. 1999;8:1837–50. ISI:000084444300008. 10620228 10.1046/j.1365-294x.1999.00773.x

[9] Frank JL, Anglin S, Carrington EM, Taylor DS, Viratos B, Southworth D. Rodent dispersal of fungal spores promotes seedling establishment away from mycorrhizal networks on *Quercus garryana*. Botany-Botanique. 2009;87:821–9. doi: 10.1139/B09-044. ISI:000270733200002.

[10] Glassman SI, Peay KG, Talbot JM, Smith DP, Chung JA, Taylor JW, et al A continental view of pine-associated ectomycorrhizal fungal spore banks: a quiescent functional guild with a strong biogeographic pattern. New Phytol. 2015;205:1619–31. doi: 10.1111/nph.13240. ISI:000349386300031. 25557275 10.1111/nph.13240

[11] Miyamoto Y, Nara K. Soil propagule banks of ectomycorrhizal fungi share many common species along an elevation gradient. Mycorrhiza. 2016;26:189–97. doi: 10.1007/s00572-015-0658-z. ISI:000372908100002. 26231215 10.1007/s00572-015-0658-z

[12] Murata M, Nagata Y, Nara K. Soil spore banks of ectomycorrhizal fungi in endangered Japanese Douglas-fir forests. Ecol Res. 2017;32:469–79.

[13] Visser S. Ectomycorrhizal Fungal Succession in Jack Pine Stands Following Wildfire. New Phytol. 1995;129:389–401. doi: 10.1111/j.1469-8137.1995.tb04309.x. ISI:A1995QR30600002.

[14] Baar J, Horton TR, Kretzer AM, Bruns TD. Mycorrhizal colonization of *Pinus muricata* from resistant propagules after a stand-replacing wildfire. New Phytol. 1999;143:409–18. doi: 10.1046/j.1469-8137.1999.00452.x. ISI:000082206200018.

[15] Huang J, Nara K, Zong K, Lian CL. Soil Propagule Banks of Ectomycorrhizal Fungi Along Forest Development Stages After Mining. Microb Ecol. 2015;69:768–77. doi: 10.1007/s00248-014-0484-4. ISI:000353295800005. 25213652 10.1007/s00248-014-0484-4

[16] Kjoller R, Bruns TD. *Rhizopogon* spore bank communities within and among California pine forests. Mycologia. 2003;95:603–13. doi: 10.2307/3761936. ISI:000185913200006. 21148969 10.1080/15572536.2004.11833064

[17] Rusca TA, Kennedy PG, Bruns TD. The effect of different pine hosts on the sampling of *Rhizopogon* spore banks in five Eastern Sierra Nevada forests. New Phytol. 2006;170:551–60. doi: 10.1111/j.1469-8137.2006.01689.x. ISI:000236738600014. 16626476 10.1111/j.1469-8137.2006.01689.x

[18] Bruns TD, Peay KG, Boynton PJ, Grubisha LC, Hynson NA, Nguyen NH, et al Inoculum potential of *Rhizopogon* spores increases with time over the first 4 yr of a 99-yr spore burial experiment. New Phytol. 2009;181:463–70. doi: 10.1111/j.1469-8137.2008.02652.x. ISI:000261792900020. 19121040 10.1111/j.1469-8137.2008.02652.x

[19] Nguyen NH, Hynson NA, Bruns TD. Stayin' alive: survival of mycorrhizal fungal propagules from 6-yr-old forest soil. Fungal Ecol. 2012;5:741–6. doi: 10.1016/j.funeco.2012.05.006. ISI:000310717800012.

[20] Ashkannejhad S, Horton TR. Ectomycorrhizal ecology under primary succession on coastal sand dunes: interactions involving *Pinus contorta*, suilloid fungi and deer. New Phytol. 2006;169:345–54. doi: 10.1111/j.1469-8137.2005.01593.x. ISI:000234482900013. 16411937 10.1111/j.1469-8137.2005.01593.x

[21] Grubisha LC, Bergemann SE, Bruns TD. Host islands within the California Northern Channel Islands create fine-scale genetic structure in two sympatric species of the symbiotic ectomycorrhizal fungus *Rhizopogon*. Mol Ecol. 2007;16(9):1811–22. doi: 10.1111/j.1365-294X.2007.03264.x. ISI:000245697000004. 17444894 10.1111/j.1365-294X.2007.03264.x

[22] Dunham SM, Mujic AB, Spatafora JW, Kretzer AM. Within-population genetic structure differs between two sympatric sister-species of ectomycorrhizal fungi, *Rhizopogon vinicolor* and *R*. *vesiculosus*. Mycologia. 2013;105(4):814–26. doi: 10.3852/12-265. ISI:000322849500003. 23709483 10.3852/12-265

[23] Abe H, Tabuchi A, Okuda Y, Matsumoto T, Nara K. Population genetics and fine-scale genetic structure of *Rhizopogon roseolus* in the Tottori sand dune. Mycoscience. 2017;58(1):14–22. doi: 10.1016/j.myc.2016.07.009. ISI:000390435900002.

[24] Mujic AB, Hosaka K, Spatafora JW. *Rhizopogon togasawariana* sp nov., the first report of *Rhizopogon* associated with an Asian species of *Pseudotsuga*. Mycologia. 2014;106:105–12. doi: 10.3852/13-055. ISI:000332429400011. 24396108 10.3852/13-055

[25] Koizumi T, Nara K. Two new species of *Rhizopogon* associated with *Pinus pumila* from Japan. Mycoscience. 2016;57(4):287–94. doi: 10.1016/j.myc.2016.04.002. ISI:000378057300008.

[26] Huang J, Nara K, Zong K, Wang J, Xue SG, Peng KJ, et al Ectomycorrhizal fungal communities associated with Masson pine (*Pinus massoniana*) and white oak (*Quercus fabri*) in a manganese mining region in Hunan Province, China. Fungal Ecol. 2014:1–10. doi: 10.1016/j.funeco.2014.01.001. ISI:000336951300001.

[27] Farjon A. Pines: drawings and descriptions of the genus Pinus. 2nd ed. Leiden; Boston: Brill; 2005. 235 p. p.

[28] Ryberg PE, Rothwell GW, Stockey RA, Hilton J, Mapes G, Riding JB. Reconsidering Relationships among Stem and Crown Group Pinaceae: Oldest Record of the Genus *Pinus* from the Early Cretaceous of Yorkshire, United Kingdom. Int J Plant Sci. 2012;173(8):917–32. doi: 10.1086/667228. ISI:000308908800006.

[29] Hao ZZ, Liu YY, Nazaire M, Wei XX, Wang XQ. Molecular phylogenetics and evolutionary history of sect. Quinquefoliae (*Pinus*): Implications for Northern Hemisphere biogeography. Mol Phylogen Evol. 2015;87:65–79. doi: 10.1016/j.ympev.2015.03.013. ISI:000353368700006. 25800283 10.1016/j.ympev.2015.03.013

[30] Frankham R, Ballou JD, Briscoe DA. Introduction to conservation genetics 2nd ed. Cambridge, UK; New York: Cambridge University Press; 2010 xxiii, 618 p. p.

[31] Ministry of Environment. Red list of Japanese threatened species 2015 [cited 2016 July]. Available from: http://ikilog.biodic.go.jp/Rdb/.

[32] Murata M, Kinoshita A, Nara K. Revisiting the host effect on ectomycorrhizal fungal communities: implications from host-fungal associations in relict *Pseudotsuga japonica* forests. Mycorrhiza. 2013;23:641–53. doi: 10.1007/s00572-013-0504-0. ISI:000325969000003. 23702643 10.1007/s00572-013-0504-0

[33] Yatoh K. Materials for the botanical study on the forest flora of the Kii Peninsula. Analysis and classification of the forest communities (in Japanese). Bull Fac Agr Mie Univ. 1958;18:105–67.

[34] Yamanaka T. Ecology of *Pseudotsuga japonica* and other coniferous forests in eastern Shikoku (in Japanese with English summary). Memoirs of the National Science Museum. 1975;8:119–36.

[35] Nara K, Nakaya H, Wu BY, Zhou ZH, Hogetsu T. Underground primary succession of ectomycorrhizal fungi in a volcanic desert on Mount Fuji. New Phytol. 2003;159:743–56. ISI:000184616400021. 10.1046/j.1469-8137.2003.00844.x 33873602

[36] Ishida TA, Nara K, Hogetsu T. Host effects on ectomycorrhizal fungal communities: insight from eight host species in mixed conifer-broadleaf forests. New Phytol. 2007;174:430–40. Epub 2007/03/29. doi: 10.1111/j.1469-8137.2007.02016.x . 17388905 10.1111/j.1469-8137.2007.02016.x

[37] White TJ, Bruns T, Lee S, Taylor J. Amplification and direct sequencing of fungal ribosomal RNA genes for phylogenetics Innis MAG D. H.; Snisky J. J.; White T. J., editor. Berkeley, CA, USA: Academic Press; 1990.

[38] Gardes M, Bruns TD. ITS primers with enhanced specificity for basidiomycetes—application to the identification of mycorrhizae and rusts. Mol Ecol. 1993;2:113–8. Epub 1993/04/01. . 8180733 10.1111/j.1365-294x.1993.tb00005.x

[39] Tedersoo L, Jairus T, Horton BM, Abarenkov K, Suvi T, Saar I, et al Strong host preference of ectomycorrhizal fungi in a Tasmanian wet sclerophyll forest as revealed by DNA barcoding and taxon-specific primers. New Phytol. 2008;180:479–90. Epub 2008/07/18. doi: 10.1111/j.1469-8137.2008.02561.x . 18631297 10.1111/j.1469-8137.2008.02561.x

[40] Bahram M, Polme S, Koljalg U, Tedersoo L. A single European aspen (*Populus tremula*) tree individual may potentially harbour dozens of *Cenococcum geophilum* ITS genotypes and hundreds of species of ectomycorrhizal fungi. FEMS Microbiol Ecol. 2011;75(2):313–20. doi: 10.1111/j.1574-6941.2010.01000.x. ISI:000285877100012. 21114502 10.1111/j.1574-6941.2010.01000.x

[41] Douhan GW, Huryn KL, Douhan LI. Significant diversity and potential problems associated with inferring population structure within the *Cenococcum geophilum* species complex. Mycologia. 2007;99:812–9. ISI:000253080600003. 18333505 10.3852/mycologia.99.6.812

[42] Colwell RK, Chao A, Gotelli NJ, Lin SY, Mao CX, Chazdon RL, et al Models and estimators linking individual-based and sample-based rarefaction, extrapolation and comparison of assemblages. Journal of Plant Ecology. 2012;5:3–21. doi: 10.1093/Jpe/Rtr044. ISI:000299103600002.

[43] Borcard D, Gillet F, Legendre P. Numerical Ecology with R. Numerical Ecology with R. 2011:1–300. doi: 10.1007/978-1-4419-7976-6. ISI:000286903500001.

[44] Anderson MJ. A new method for non-parametric multivariate analysis of variance. Austral Ecol. 2001;26:32–46. doi: 10.1111/j.1442-9993.2001.01070.pp.x. ISI:000167002000004.

[45] Oksanen JBG, Kindt R, Legendre P, O' Hara B, Simpson GL, Solymos P, et al Package 'vegan' community ecology package. R package version 1.17–2. 2010.

[46] Buscardo E, Rodriguez-Echeverria S, Martin MP, De Angelis P, Pereira JS, Freitas H. Impact of wildfire return interval on the ectomycorrhizal resistant propagules communities of a Mediterranean open forest. Fungal Biology. 2010;114:628–36. doi: 10.1016/j.funbio.2010.05.004. ISI:000280928100004. 20943174 10.1016/j.funbio.2010.05.004

[47] Kipfer T, Moser B, Egli S, Wohlgemuth T, Ghazoul J. Ectomycorrhiza succession patterns in *Pinus sylvestris* forests after stand-replacing fire in the Central Alps. Oecologia. 2011;167:219–28. Epub 2011/04/07. doi: 10.1007/s00442-011-1981-5 . 21468664 10.1007/s00442-011-1981-5

[48] Glassman SI, Levine CR, DiRocco AM, Battles JJ, Bruns TD. Ectomycorrhizal fungal spore bank recovery after a severe forest fire: some like it hot. Isme J. 2016;10(5):1228–39. doi: 10.1038/ismej.2015.182. ISI:000374377200019. 26473720 10.1038/ismej.2015.182 PMC5029211

[49] Kanetani S, Gyokusen K, Ito S, Saito A. The distribution pattern of *Pinus armandii* Franch. var. *amamiana* Hatusima around Mt. Hasha-dake in Yaku-shima Island (in Japanese). J Jpn For Soc. 1997;79:160–3.

[50] Kanetani S, Gyokusen K, Ito S, Saito A. The floristic composition of *Pinus armandii* var. *amamiana* forests on Yaku-shima Island, southwestern Japan (in Japanese). Res Bull Kagoshima Univ For. 2010;37:49–61.

[51] Murata M, Nara K. Ectomycorrhizal fungal communities at different soil depths in a forest dominated by endangered *Pseudotsuga japonica* (in Japanese). J Jpn For Soc. 2017.

[52] Izzo A, Canright M, Bruns TD. The effects of heat treatments on ectomycorrhizal resistant propagules and their ability to colonize bioassay seedlings. Mycol Res. 2006;110:196–202. doi: 10.1016/j.mycres.2005.08.010. ISI:000235919200008. 16387485 10.1016/j.mycres.2005.08.010

[53] Peay KG, Garbelotto M, Bruns TD. Spore heat resistance plays an important role in disturbance-mediated assemblage shift of ectomycorrhizal fungi colonizing *Pinus muricata* seedlings. J Ecol. 2009;97:537–47. doi: 10.1111/j.1365-2745.2009.01489.x. ISI:000265035400016.

[54] Kipfer T, Egli S, Ghazoul J, Moser B, Wohlgemuth T. Susceptibility of ectomycorrhizal fungi to soil heating. Fungal Biology. 2010;114:467–72. doi: 10.1016/j.funbio.2010.03.008. ISI:000279389000009. 20943157 10.1016/j.funbio.2010.03.008

[55] Grubisha LC, Trappe JM, Molina R, Spatafora JW. Biology of the ectomycorrhizal genus Rhizopogon. VI. Re-examination of infrageneric relationships inferred from phylogenetic analyses of ITS sequences. Mycologia. 2002;94:607–19. ISI:000177089800006. 21156534 10.1080/15572536.2003.11833189

[56] Wen ZG, Murata MS, Xu ZY, Chen YH, Nara K. Ectomycorrhizal fungal communities on the endangered Chinese Douglas-fir (*Pseudotsuga sinensis*) indicating regional fungal sharing overrides host conservatism across geographical regions. Plant Soil. 2015;387:189–99. doi: 10.1007/s11104-014-2278-3. ISI:000348318100014.

[57] Watanabe A, Shiraishi S. Classification of Haploxylon distributed in Asia using DNA sequences (in Japanese). Transactions of Kyushu Branch of the Jpn For Soc. 1996;49:57–8.

[58] Kanetani S, Kawahara T, Kanazashi A, Yoshimaru H. Diversity and conservation of genetic resources of an endangered Japanese five-needle pine species, Pinus armandii Franch. var. amamiana (Koidz.) Hatusima. USDA Forest Service Proceedings. 2004;RMRS-P-32:188–91.

[59] Molina R, Trappe JM, Grubisha LC, Spatafora JW. *Rhizopogon* In: J.W.G. C, S.M. C, editors. Ectomycorrhizal Fungi: Key Genera in Profile. Berlin: Springer Verlag; 1999 p. 129–61.

[60] Peay KG, Schubert MG, Nguyen NH, Bruns TD. Measuring ectomycorrhizal fungal dispersal: macroecological patterns driven by microscopic propagules. Mol Ecol. 2012;21:4122–36. doi: 10.1111/j.1365-294X.2012.05666.x. ISI:000306897500019. 22703050 10.1111/j.1365-294X.2012.05666.x

[61] Hynson NA, Merckx VSFT, Perry BA, Treseder KK. Identities and distributions of the co-invading ectomycorrhizal fungal symbionts of exotic pines in the Hawaiian Islands. Biol Invasions. 2013;15(11):2373–85. doi: 10.1007/s10530-013-0458-3. ISI:000325555300003.

[62] Nara K. Spores of ectomycorrhizal fungi: ecological strategies for germination and dormancy. New Phytol. 2009;181:245–8. doi: 10.1111/j.1469-8137.2008.02691.x. ISI:000261792900002. 19121026 10.1111/j.1469-8137.2008.02691.x

[63] Wu QX, Mueller GM, Lutzoni FM, Huang YQ, Guo SY. Phylogenetic and biogeographic relationships of eastern Asian and eastern north American disjunct *Suillus* species (Fungi) as inferred from nuclear ribosomal RNA ITS sequences. Mol Phylogen Evol. 2000;17(1):37–47. doi: 10.1006/mpev.2000.0812. ISI:000089952700005. 11020303 10.1006/mpev.2000.0812

[64] Hirose D, Shirouzu T, Tokumasu S. Host range and potential distribution of ectomycorrhizal basidiomycete *Suillus pictus* in Japan. Fungal Ecol. 2010;3(3):255–60.

[65] Liao HL, Chen Y, Vilgalys R. Metatranscriptomic Study of Common and Host-Specific Patterns of Gene Expression between Pines and Their Symbiotic Ectomycorrhizal Fungi in the Genus *Suillus*. PLoS Genet. 2016;12(10). ARTN e1006348 doi: 10.1371/journal.pgen.1006348. ISI:000386683300017. 27736883 10.1371/journal.pgen.1006348 PMC5065116

[66] Kikuchi J, Futai K. Spatial distribution of sporocarps and the biomass of ectomycorrhizas of *Suillus pictus* in a Korean pine (*Pinus koraiensis*) stand. J For Res. 2003;8(1):17–25.

[67] Zhang R, Mueller MM, Shi X, Liu P. Two new species in the *Suillus spraguei* complex from China. Mycologia. 2017;109(2):296–307. doi: 10.1080/00275514.2017.1305942 28463625 10.1080/00275514.2017.1305942

[68] Hayashi Y. Taxonomical and phytogeographical study of Japanese conifers. Tokyo: Norin; 1960.

