# Supplementary figures and images for: Ectomycorrhizal fungal communities in endangered Pinus amamiana forests
Source: PLoS One. 2017 Dec 19;12(12):e0189957. doi: 10.1371/journal.pone.0189957 (PMC5736215; doi:10.1371/journal.pone.0189957)

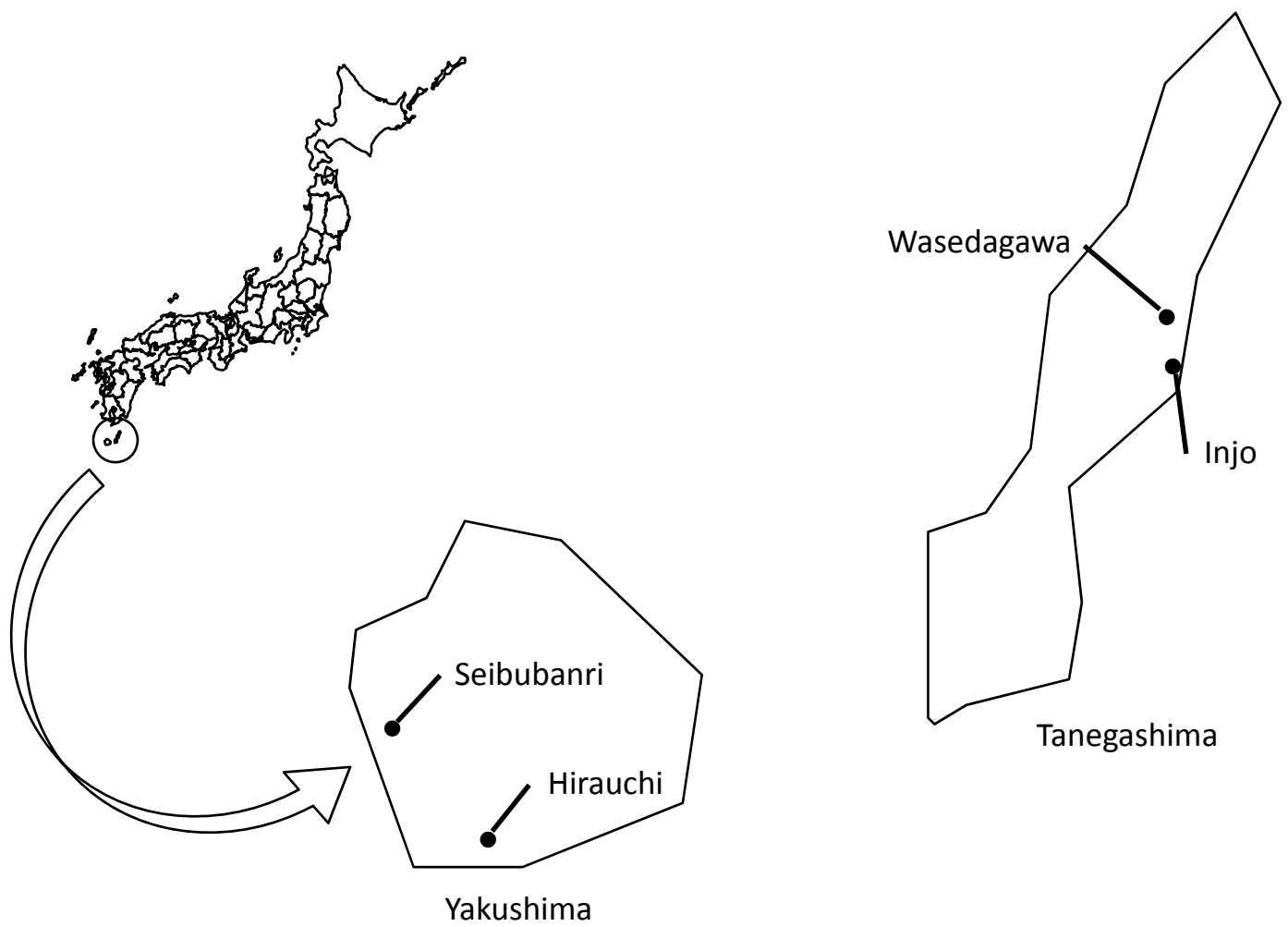

S1 Appendix. Location of four study sites in Japan

Supplement: S1 Appendix — (PDF) [file pone.0189957.s001.pdf]
