# Supplementary material for: Ectomycorrhizal fungal communities in endangered Pinus amamiana forests
Source: PLoS One. 2017 Dec 19;12(12):e0189957. doi: 10.1371/journal.pone.0189957 (PMC5736215; doi:10.1371/journal.pone.0189957)
Supplement: S2 Appendix — (PDF) [file pone.0189957.s002.pdf]

S2 Appendix. Air-dried periods, Growth periods and number of sample of each tree species in bioassay experiment

Seedlings species	Air-dried periods (month)	Growth periods (month)	Number of sample
Pinus amamiana	2	6	24
	8	6	22
P. parviflora	2-3	6-8	79
P. densiflora	2	6	79
Castanopsis sieboldii	3	9	79
