# Supplementary material for: Ectomycorrhizal fungal communities in endangered Pinus amamiana forests
Source: PLoS One. 2017 Dec 19;12(12):e0189957. doi: 10.1371/journal.pone.0189957 (PMC5736215; doi:10.1371/journal.pone.0189957)
Supplement: S3 Appendix — (PDF) [file pone.0189957.s003.pdf]

S3 Appendix. Blast results of ectomycorrhizal fungal species identified in this study using UNITE database.

	Best UNITE match				
ECM fungi	Accession No.	Taxon name	Identities (%)	Score (Bit)	E Value
Amanita sp.1	KP711844	*Amanita*	96	884	0.0
Amanita sp.2	AB922858	*Amanita*	99	1139	0.0
Amanita sp.3	AB981985	Fungi	99	1234	0.0
Amanita sp.4	UDB033611	*Amanita virgineoides*	–	334	1.00E−90
Amanitaceae sp.1	KP313580	*Amanita frostiana*	86	448	2.00E−124
Atheliaceae sp.1	AB456674	*Amphinema*	99	993	0.0
Austroboletus sp.1	UDB032685	*Austroboletus fusisporus*	–	1079	0.0
Boletellus aurocontextus	AB989014	Boletellus	99	1436	0.0
Boletus sp.1	AB973752	*Boletus*	99	1144	0.0
Boletus sp.2	HM347643	*Boletus erythropus*	82	610	4.00E−173
Boletus sp.3	UDB024501	*Boletus*	–	1146	0.0
Boletaceae sp.1	AB972824	Boletaceae	100	789	0.0
Boletaceae sp.2	UDB022819	*Boletus erythropus*	82	728	0.0
Boletaceae sp.3	KY826191	Boletales	92	1025	0.0
Boletaceae sp.4	UDB013160	Boletaceae	95	407	3.00E−112
Boletaceae sp.5	KC551993	Boletaceae	99	1380	0.0
Boletaceae sp.6	AB973728	*Tylopilus*	99	1368	0.0
Boletaceae sp.7	UDB032623	*Tylopilus*	–	1067	0.0
Boletaceae sp.8	KM595001	Boletales	91	890	0.0
Boletaceae sp.9	JQ991917	Boletaceae	100	1034	0.0
Boletaceae sp.10	JF273511	*Xerocomus*	99	1200	0.0
Cantharellaceae sp1	KT200524	*Hydnum*	93	719	0.0
Cenococcum geophilum	–	–	–	–	–
Ceratobasidium sp.1	AB303058	Ceratobasidiaceae	99	1083	0.0
Ceratobasidium sp.2	JQ991676	Ceratobasidiaceae	97	951	0.0
Ceratobasidiaceae sp.1	AB605643	Ceratobasidiaceae	100	1056	0.0
Ceratobasidiaceae sp.2	AB605649	Ceratobasidiaceae	95	982	0.0
Clavulina sp.1	JF273519	Membranomyces	100	1029	0.0
Clavulina sp.2	JQ991682	Clavulinaceae	99	848	0.0
Clavulinaceae sp.1	UDB017244	Clavulinaceae	88	782	0.0
Clavulinaceae sp.2	AB807913	Clavulinaceae	99	1164	0.0
Clavulinaceae sp.3	AB807910	Clavulinaceae	100	682	0.0
Clavulinaceae sp.4	KC876295	Clavulinaceae	86	719	0.0
Clavulinaceae sp.5	AB848424	Clavulinaceae	100	1070	0.0
Clavulinaceae sp.6	KC876295	Clavulinaceae	89	286	6.00E−76
Clavulinaceae sp.7	UDB007628	Clavulinaceae	88	753	0.0
Coltricia sp.1	KU360702	*Coltriciella subglobosa*	95	423	4.00E−117
Cortinarius sp.1	UDB013282	*Cortinarius*	95	1162	0.0
Cortinarius sp.2	AB848438	*Cortinarius*	99	1094	0.0
Cortinarius sp.3	UDB024556	*Cortinarius*	–	1067	0.0
Cortinarius sp.4	AB973753	*Cortinarius*	99	1402	0.0
Cortinarius sp.5	AB982000	Fungi	99	908	0.0
Craterellus sp.1	JQ991672	Cantharellaceae	94	354	0.0
Craterellus sp.2	AB973729	*Craterellus tubaeformis*	98	890	0.0
Elaphomyces sp.1	JQ991717	Elaphomycetaceae	94	758	0.0
Elaphomyces sp.2	EU597039	*Elaphomyces*	97	681	0.0
Elaphomycetaceae sp.1	JQ991901	Aspergillaceae	98	578	1.00E−163
Entoloma sp.1	UDB031080	*Entoloma rhodocylix*	–	942	0.0
Hydnellum sp.1	UDB014154	*Hydnellum*	99	1059	0.0
Hydnellum sp.2	KF617227	*Sarcodon*	86	666	0.0

	Best UNITE match				
ECM fungi	Accession No.	Taxon name	Identities (%)	Score (Bit)	E Value
Hydnum sp.1	KU612573	*Hydnum*	99	935	0.0
Hydnum sp.2	AB906676	*Hydnum*	100	1029	0.0
Hydnum sp.3	AB251813	*Hydnum*	100	710	0.0
Hymenochaetaceae sp.1	JQ991687	Hymenochaetaceae	87	834	0.0
Hymenochaetaceae sp.2	KM594897	*Coltriciella*	90	926	0.0
Inocybe sp.1	AM882711	*Inocybe subexilis*	94	814	0.0
Laccaria vinaceoavellanea	JN942811	*Laccaria vinaceoavellanea*	99	852	0.0
Lactarius sp.1	AB777482	*Lactarius*	99	1126	0.0
Lactarius sp.2	LC096473	*Lactarius*	99	1177	0.0
Lactarius sp.3	JQ991640	*Lactarius*	99	1076	0.0
Lactarius sp.4	GQ268638	*Lactarius*	98	1112	0.0
Lactarius sp.5	AB973742	*Lactarius*	99	1020	0.0
Lactarius sp.6	JQ991763	*Lactarius*	94	820	0.0
Pezizaceae sp.1	JN102406	Pezizaceae	94	845	0.0
Pezizaceae sp.2	JQ991767	Pezizaceae	95	565	7.00E−160
Pezizaceae sp.3	AB571493	Pezizaceae	100	962	0.0
Phylloporus sp.1	GU328591	Boletaceae	90	1180	0.0
Phylloporus sp.2	UDB032523	*Phylloporus*	−	1287	0.0
Phylloporus sp.3	JQ967243	*Phylloporus brunneiceps*	79	654	0.0
Rhizopogon sp.1	LC096919	*Rhizopogon*	93	1135	0.0
Rhizopogon sp.2	AB923020	*Rhizopogon*	99	506	5.00E−142
Rhizopogon sp.3	AB253521	*Rhizopogon*	98	919	0.0
Rossbeevera griseovelutina	KC551986	*Rossbeevera griseovelutina*	99	904	0.0
Russula sp.1	AB507012	*Russula*	99	1166	0.0
Russula sp.2	JX556185	*Russula*	99	1077	0.0
Russula sp.3	JX987768	*Russula*	99	1086	0.0
Russula sp.4	LC096863	*Russula*	99	1090	0.0
Russula sp.5	JQ991802	*Russula*	99	1067	0.0
Russula sp.6	AB507006	*Russula*	100	1088	0.0
Russula sp.7	LC096934	*Russula*	96	1169	0.0
Russula sp.9	UDB031147	*Russula melliolens*	93	729	0.0
Russula sp.10	AB594957	*Russula*	99	1032	0.0
Russula sp.11	JX178489	*Russula roseopileata*	90	715	0.0
Russula sp.12	AB629011	*Russula*	99	1021	0.0
Russula sp.13	AB636110	*Lactifluus*	99	935	0.0
Russula sp.14	LC096811	*Russula*	100	1297	0.0
Russula sp.15	KJ769295	*Russula*	99	1296	0.0
Russula sp.16	UDB013176	*Russula*	96	812	0.0
Russula sp.17	AB291762	*Russula densifolia*	99	1074	0.0
Russula sp.18	JQ991823	*Russula*	99	1041	0.0
Russula sp.19	UDB025322	*Russula*	99	1083	0.0
Russula sp.20	JQ991790	*Russula*	99	1085	0.0
Russula sp.21	AB769909	*Russula*	95	946	0.0
Russula sp.23	FJ454902	*Russula*	99	1067	0.0
Russula sp.24	UDB013176	*Russula*	98	504	2.00E−141
Russula sp.25	HE814113	*Russula*	99	948	0.0
Russulaceae sp.1	UDB013177	*Russula*	95	973	0.0
Russulaceae sp.2	KM594995	*Russula*	95	812	0.0
Sarcodon sp.1	KR673603	*Sarcodon scabrosus*	95	919	0.0
Sistotrema sp.1	JX561240	*Sistotrema*	94	812	0.0
Suillus bovinus	AB571499	*Suillus bovinus*	99	1274	0.0

	Best UNITE match				
ECM fungi	Accession No.	Taxon name	Identities (%)	Score (Bit)	E Value
Thelephoraceae sp.1	UDB013763	Thelephoraceae	98	1213	0.0
Thelephoraceae sp.2	JF273549	*Tomentella*	99	1088	0.0
Thelephoraceae sp.3	KM403070	Thelephoraceae	96	1025	0.0
Thelephoraceae sp.4	FJ210778	Thelephoraceae	95	944	0.0
Thelephoraceae sp.5	AB854719	Thelephoraceae	98	1021	0.0
Thelephoraceae sp.6	UDB008037	Thelephoraceae	97	1242	0.0
Thelephoraceae sp.7	JX456961	Thelephoraceae	99	1090	0.0
Thelephoraceae sp.8	JX456812	Thelephoraceae	95	953	0.0
Tricholoma sp.1	UDB023544	*Tricholoma ustaloides*	98	1067	0.0
Tuber sp.1	AB285533	*Tuber*	98	901	0.0
