# Supplementary material for: Ectomycorrhizal fungal communities in endangered Pinus amamiana forests
Source: PLoS One. 2017 Dec 19;12(12):e0189957. doi: 10.1371/journal.pone.0189957 (PMC5736215; doi:10.1371/journal.pone.0189957)
Supplement: S4 Appendix — Stress = 0.142. White, gray and black symbols indicate the communities on Fagaceae, P. amamiana and Tsuga sieboldii, respectively. Circles, squares, diamonds and triangles represent communities at sites 1, 2, 3 and 4, respectively. The effects of both host and site were significant (Adonis, P<0.01) after confirming data variance among the groups was not significant (Betadisper, P>0.05). (PDF) [file pone.0189957.s004.pdf]

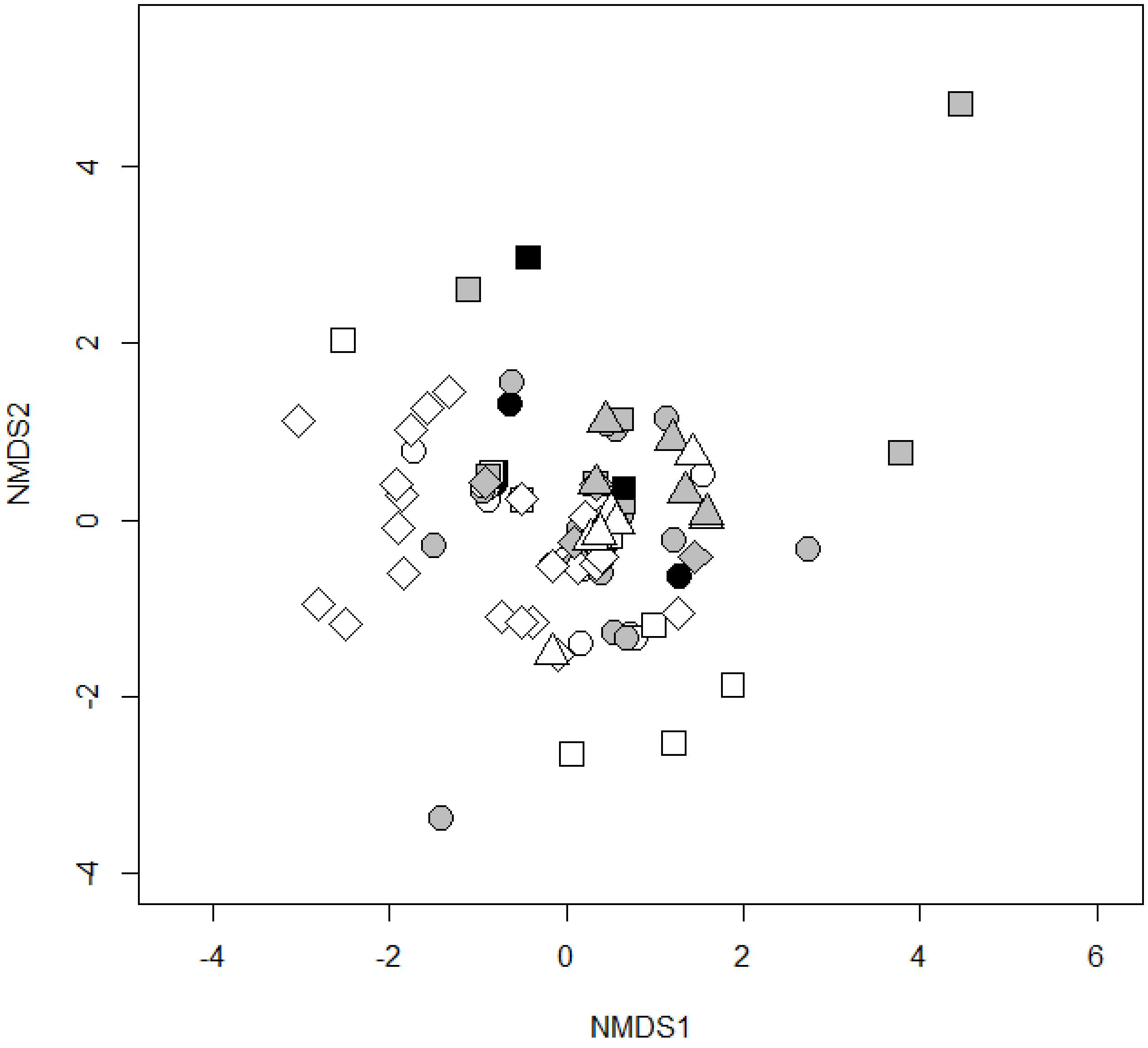

S4 Appendix. Non-metric multidimensional scaling (NMDS) depicting ECM fungal communities of resident trees in four endangered *Pinus amamiana* forests based on individual soil samples. Stress = 0.142. White, gray and black symbols indicate the communities on Fagaceae, *P. amamiana* and *Tsuga sieboldii* , respectively. Circles, squares, diamonds and triangles represent communities at sites 1, 2, 3 and 4, respectively. The effects of both host and site were significant (Adonis, P<0.01) after confirming data variance among the groups was not significant (Betadisper, P>0.05).
