# Supplementary material for: Ectomycorrhizal fungal communities in endangered Pinus amamiana forests
Source: PLoS One. 2017 Dec 19;12(12):e0189957. doi: 10.1371/journal.pone.0189957 (PMC5736215; doi:10.1371/journal.pone.0189957)
Supplement: S5 Appendix — (PDF) [file pone.0189957.s005.pdf]

S4 Appendix . Summary of bioassays using soil from three endangered *Pinus amamiana* forests

Host	*Pinus amamiana*			*P. parviflora*			*P. densiflora*			*Castanopsis sieboldii*		
Site	1	2	3	1	2	3	1	2	3	1	2	3
Total seedlings	23	6	17	26	21	32	26	21	32	26	21	32
Surviving seedlings	20	6	17	20	17	27	26	20	31	24	20	28
Ectomycorrhizal (ECM) seedlings	20	6	17	12	15	25	17	11	30	15	16	27
DNA samples (ECM tips)	83	31	95	50	66	128	60	43	152	50	47	92
Sequences obtained	79	31	95	48	64	128	59	43	143	50	47	91
Detected ECM fungal species	5	2	2	4	2	2	6	3	5	4	1	5
