# Supplementary material for: Ectomycorrhizal fungal communities in endangered Pinus amamiana forests
Source: PLoS One. 2017 Dec 19;12(12):e0189957. doi: 10.1371/journal.pone.0189957 (PMC5736215; doi:10.1371/journal.pone.0189957)
Supplement: S6 Appendix — Each symbol represents a community in each host per site. Stress = 0.134. White and black symbols indicate the communities assayed with resident trees and soil propagule banks, respectively. (PDF) [file pone.0189957.s006.pdf]

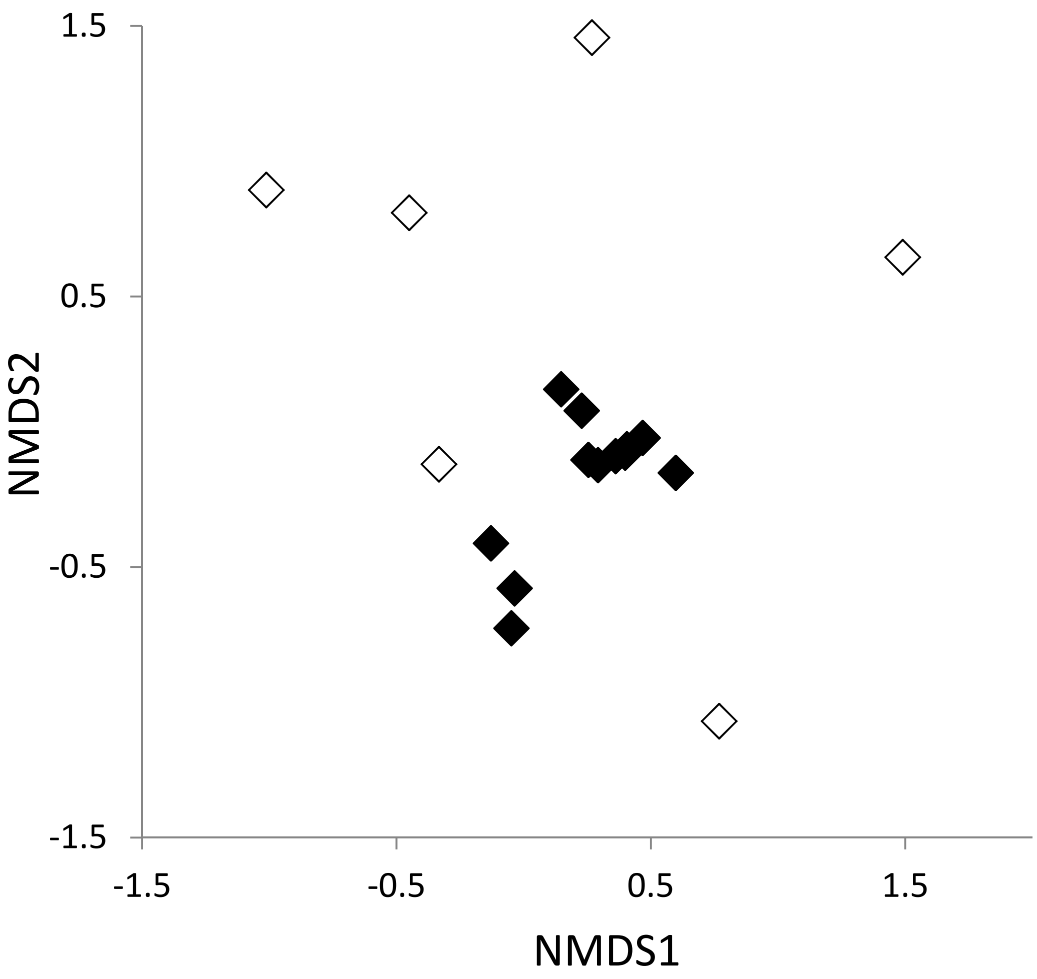

S4 Appendix. Comparison of ectomycorrhizal fungal communities between soil propagule banks and resident trees in endangered *Pinus amamiana* forests using non-metric dimensional scaling (NMDS). Each symbol represents a community in each host per site. Stress = 0.134. White and black symbols indicate the communities assayed with resident trees and soil propagule banks, respectively.
