# Supplementary material for: Ectomycorrhizal fungal communities in endangered Pinus amamiana forests
Source: PLoS One. 2017 Dec 19;12(12):e0189957. doi: 10.1371/journal.pone.0189957 (PMC5736215; doi:10.1371/journal.pone.0189957)
Supplement: S7 Appendix — Significant probability between Rhizopogon sp.1 and Control of leaf number and tree height by T test was p = 0.08 and p = 0.06, respectively. The bar shows the standard error. (PDF) [file pone.0189957.s007.pdf]

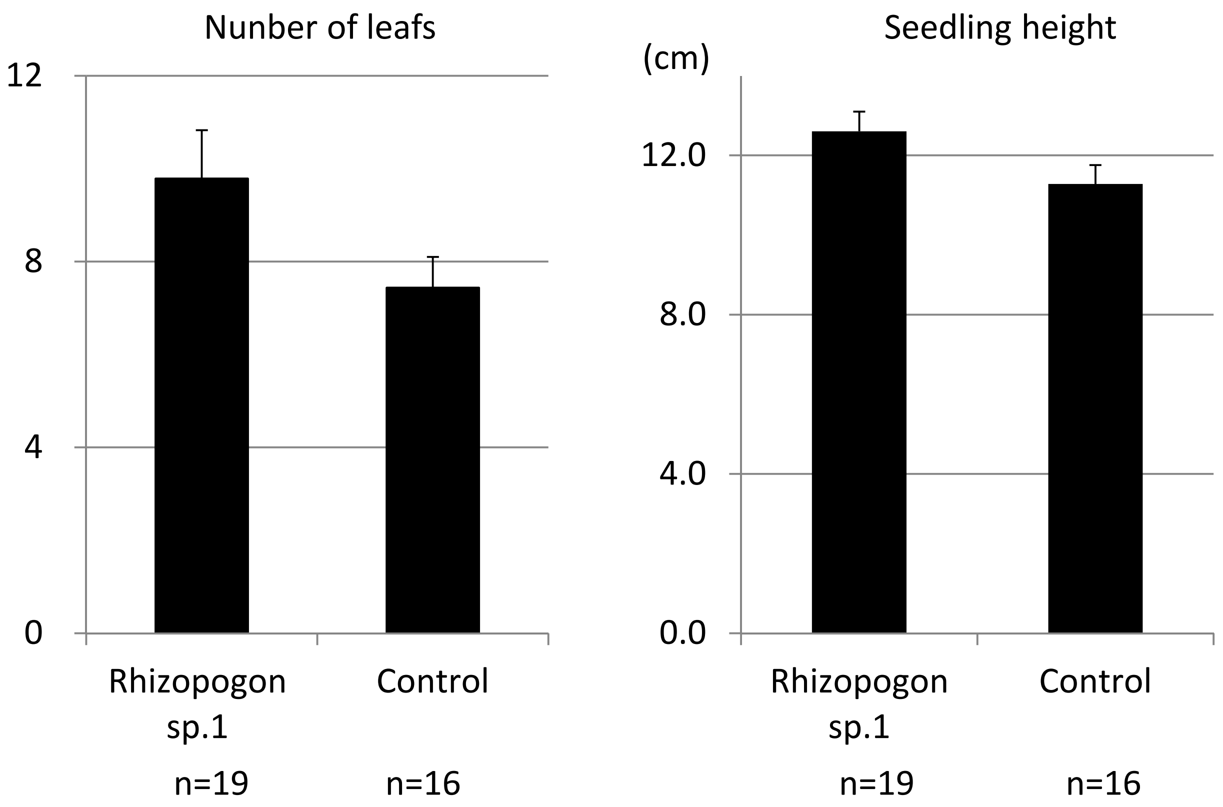

S5 Appendix. In the bioassay experiment different from this study (unpublished data), the number of leaves and tree height of seedlings infected with *Rhizopogon* sp.1 and seedlings not infected with ECM fungi (Control) grown for 1 year. Significant probability between *Rhizopogon* sp.1 and Control of leaf number and tree height by T test was p = 0.08 and p = 0.06, respectively. The bar shows the standard error.
