# Supplementary material for: Ectomycorrhizal fungal communities in endangered Pinus amamiana forests
Source: PLoS One. 2017 Dec 19;12(12):e0189957. doi: 10.1371/journal.pone.0189957 (PMC5736215; doi:10.1371/journal.pone.0189957)
Supplement: S8 Appendix — (PDF) [file pone.0189957.s008.pdf]

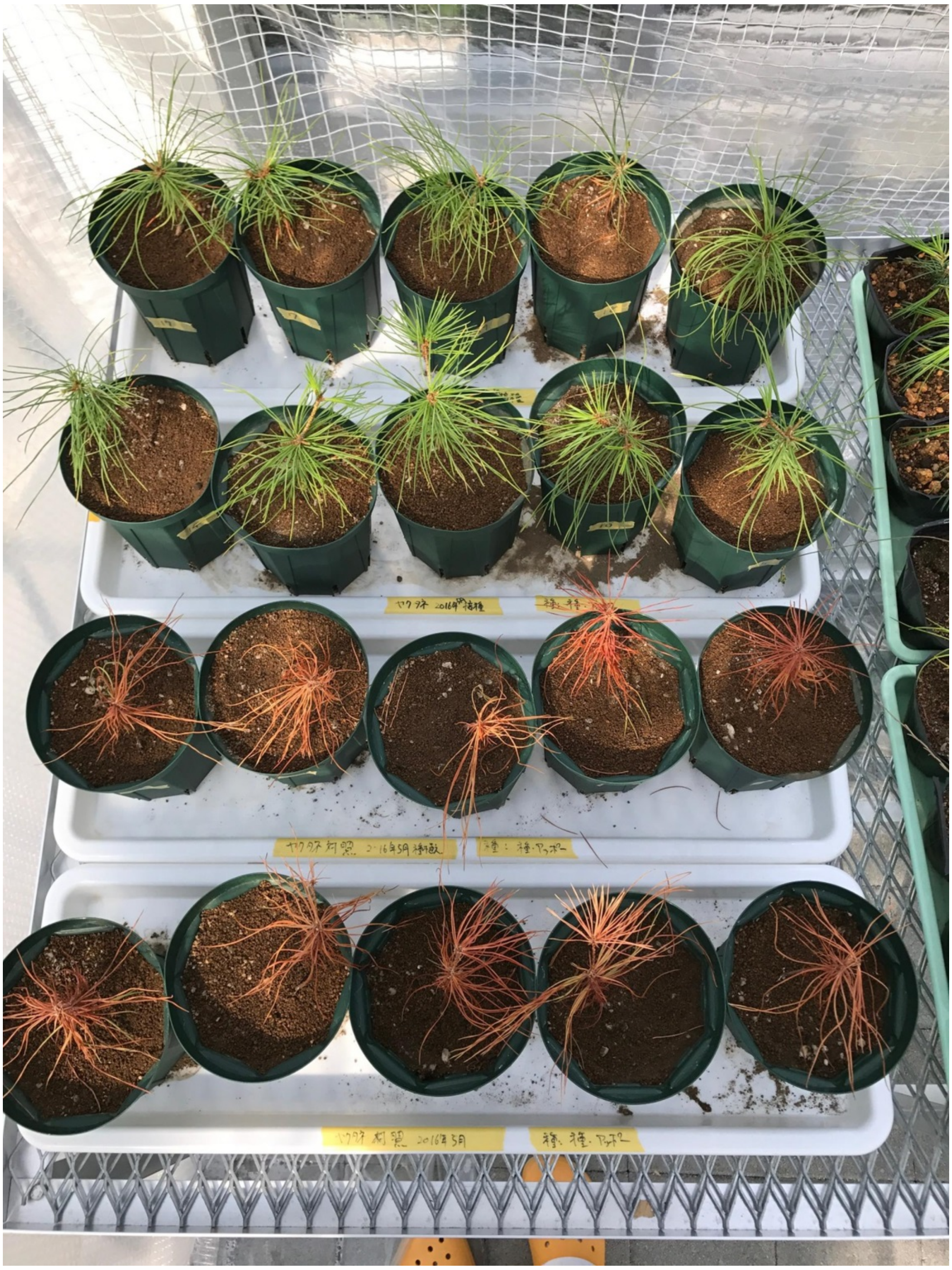

S6 Appendix. From a bioassay experiment separate from this study (unpublished data), transplantation of seedlings infected with *Rhizopogon* sp.1 (upper) and uninfected seedlings (lower) to new pots after 1 year of growth.
